# The dim light melatonin onset across ages, methodologies, and sex and its relationship with morningness/eveningness

**DOI:** 10.1093/sleep/zsad033

**Published:** 2023-02-17

**Authors:** David J Kennaway

**Affiliations:** Robinson Research Institute and Adelaide School of Medicine, University of Adelaide, Adelaide, South Australia, Australia

**Keywords:** Circadian, phase, sleep disorders, DSWPD, melatonin onset, chronotype

## Abstract

The onset of melatonin secretion, the dim light melatonin onset (DLMO), is a tool for determining the phase of the circadian timing system. Although small studies have investigated the impacts of age and methods of calculating DLMO, there is no DLMO reference range. In the current study, the saliva DLMO from 3579 participants from 121 published studies and plasma DLMO from 818 healthy controls from 31 studies (aged 3–73 years) were analyzed. In a subset of 53 papers (1749 participants), individual saliva DLMO and Morningness Eveningness Questionaire (MEQ) scores were obtained from authors or mined from publications and a reference range was constructed. Saliva DLMO was earliest in children to 10 years of age and latest around 20 years of age and thereafter advanced with age by 30 min in the oldest participants. Melatonin assay methods and DLMO calculation methods had little effect on the determination of the DLMO. Saliva DLMO was correlated (*p* < 0.001) with the MEQ score; lower MEQ scores were associated with later DLMO. MEQ scores increased with age, reflecting a tendency toward morningness. An evaluation of 14 saliva DLMO studies of clinically diagnosed patients living with delayed sleep–wake phase disorder (mean ages 20 to 31 years) revealed mean saliva DLMO within the reference range albeit at the late extreme. Peak plasma melatonin levels from 179 studies of healthy participants revealed a high degree of variability within studies and age groups, but only a small decline between the 20 and 50 years and lowest levels after 70 years.

## Graphical Abstract

**Figure:**
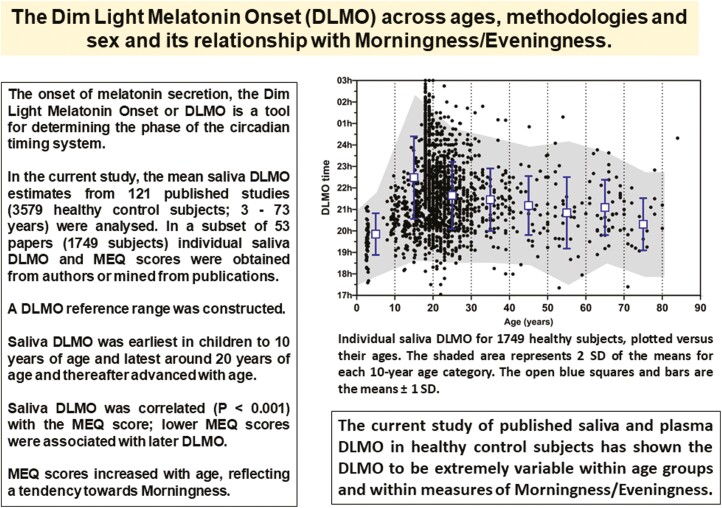

**Statement of Significance**

Although the dim light melatonin onset (DLMO) measurements have been made for many years in both research and clinical settings, there is no clear consensus about changes with age, the impact of assay methods, or the procedure used to calculate DLMO. It is also not clear how DLMO correlates with MEQ scores, with many studies finding low correlations. In this study through literature searches and data sharing, the first reference range for saliva DLMO across age groups has been prepared, together with the association between DLMO and MEQ in more than 1700 healthy participants. DLMO in patients with DSWPD was fitted into this reference range and provides strong evidence for a large proportion of these patients having normal DLMO. This study provides a rationale for DLMO to be included in the assessment of patients reporting to clinicians with DSWPD. The study also shows that assay methods and DLMO calculation procedures do not have a major impact on DLMO.

## Introduction

From the time melatonin was discovered as a product of the pineal gland, it has been recognized that its synthesis is low during the daylight and high during the night in rats [1, 2] and humans [3], indeed in most species. Since its discovery, there have been considerable advances in our knowledge on the pathways and mechanisms controlling melatonin production. In brief, light detected by the retina during the day via melanopsin-containing cells actively suppresses suprachiasmatic nucleus (SCN) signaling to the pineal gland via the superior cervical ganglion and its sympathetic input to the pineal gland. The endogenous rhythmicity of the SCN is also synchronized by the light/dark cycle. In the early evening with the decrease in light intensity and alterations in SCN cell activity, excitatory signals are sent via the Superior Cervical Ganglion (SCG) to initiate the transcription/translation of Aryl Alkylamine N-Acetyl Transferase (AANAT), the rate-limiting enzyme in the melatonin synthesis pathway. From studies conducted in humans kept in continuous dim light, abnormally short (20 h days) or long (28 h days), or totally blind individuals it was found that the SCN-driven core body temperature rhythm persists with the average of the “free-running” period greater than 24 hours [4].

Measurement of the start of melatonin production under conditions that minimized the acute effects of light promised to be useful in determining the “state” of the SCN rhythm compared with core body temperature measurement because the latter is intrusive, cumbersome, and masked by sleep [5]. Melatonin measurement was considered to have particular value in studies on sleep disorders because it was clear that the SCN was also intimately involved in sleep timing. There were some important technical challenges in putting this approach into practice. The first was obviously to develop assays for melatonin that were sensitive *and* specific for melatonin. In the early human studies, melatonin was found to circulate in the blood at night at low concentrations (<100 pg/mL). This provided some serious methodological issues to overcome which have been discussed in depth recently [6–10]. Nevertheless, in 1989, it was reported that measurement of plasma melatonin using a sensitive Gas Chromatography Mass Spectrometry (GC-MS) assay could be used to determine the circadian phase of humans [11]. Then, following the availability of sensitive radioimmunoassays for plasma melatonin, but especially those that could be used to reliably measure melatonin in saliva [12, 13] and their subsequent commercial availability (Buhlmann Laboratories (now Novolytix GmbH), Stockgrand Ltd. (no longer in business) and IBL International GmbH, the approach became more widely accessible.

More than 30 years ago, Al Lewy proposed the concept of the “Dim Light Melatonin Onset” or “DLMO” as a means of determining the phase of the circadian timing system [11]. At that time, plasma melatonin analysis was the common matrix used for melatonin assay and his laboratory used a sensitive (1 pg/mL) GC-MS assay that consistently measured daytime melatonin levels of 1–2 pg/mL. Participants avoided light exposure of >50 lux for at least 1–2 h prior to their nightly onset of melatonin production and throughout the blood- drawing. In his laboratory’s studies, the DLMO was defined as the first interpolated point above 10 pg/mL that continued to rise. Plasma DLMO assessments were used to address questions around the phase shifting of rhythms with light and melatonin and its impacts on sleep in subsequent years. It is beyond the scope of this study to address these. What is obvious about the studies is that because the plasma DLMO required frequent blood draws, all the studies had to take place in clinical research centers; they were thus expensive, time-consuming, and limited in sample sizes. The use of blood draws, which can proceed with the participant undisturbed and asleep does, however, allows researchers to assess other characteristics of melatonin secretion such as peak levels and the timing of the cessation of melatonin production, the Dim Light Melatonin Off (DLMOff) [14].

With the discovery that melatonin can be detected in saliva [15, 16], this matrix has become popular for melatonin studies since it provides noninvasive access to secreted melatonin. The melatonin in saliva is considered to represent the free, unbound plasma melatonin levels [17]. Consequently, the levels of saliva melatonin are approximately 30% of the total plasma melatonin concentration. Saliva melatonin measurements, however, should not be used to infer levels of melatonin production by the pineal gland as there is a considerable variability in the binding between individuals [9, 18, 19]. In a consensus paper on measurement of melatonin in humans [20], some guidelines for saliva sampling were proposed; samples should be “taken every 30 to 60 min under dim light (<30 lux) for at least 1 h prior to and throughout the expected rise in melatonin and subjects should follow instructions to avoid contamination of samples with food particles, food dye, or blood.” The most frequently used ways of determining DLMO were stated as “an absolute threshold in the range of 2 to 10 pg/mL, a threshold calculated at 2 SD above the average baseline and a visual estimate of the point of change from baseline to rising levels.” A further approach for determining phase when an overnight or 24-h melatonin profile is available involves calculation of a “percentage of maximum levels (e.g. 20%, 25%, or 50% of maximum) on the rising phase, the midpoint between the rising and declining phases, or the acrophase determined from a cosine curve that has been fit to the raw melatonin data” [20].

Any study involving melatonin measurement must control for the influence of certain drugs on melatonin metabolism, especially drugs metabolized by CYP1A2 and CYP2C19 [21]. Other potential confounders that might be encountered in human studies have been discussed in a previous review [9] and include various other drugs, not changing posture during the collection period, and not using citric acid or similar to stimulate saliva flow. Provided that there is compliance to scheduled sample collection times, home collection of saliva is often used and a recent study has highlighted the use of a protocol that can ensure good quality collections [22].

DLMO is used as a marker of circadian phase, predominantly in relation to sleep timing. Indeed, it has long been used to monitor treatments to correct the timing of sleep in delayed sleep–wake phase disorder (DSWPD) and advance sleep phase disorder, and non-24-h sleep disorder in research settings. It has been proposed that DLMO should be measured before treatments of circadian rhythm disorders [23, 24], but its use is not universally accepted in clinical practice settings [25, 26]. However, as pointed out by Culnan et al. [25], saliva DLMO could be useful in differentially diagnosing DSWPD from other conditions and identifying patients who met ICSD-2 criteria but do not have a later DLMO [27]. Morning light exposure, chronotherapy, and melatonin administration are used in the treatment of DSWPD [28].

Melatonin has soporific [29] as well as chronobiotic properties and administration can advance the timing of its own endogenous rhythm [30, 31], that is, advance the DLMO time and may have efficacy in the treatment of DSWPD [25, 26]. Melatonin is widely available as a “dietary supplement” without prescription in the United States and some other jurisdictions with the implicit assumption that it will help alleviate insomnia. In countries that restrict the sale of melatonin either by making it prescription-only or by requiring pharmacist advice, there are several melatonin formulations being marketed including prolonged-release formulations [32]. One prominent formulation, Circadin (Neurim Pharmaceuticals), is used “for the short-term treatment of primary insomnia characterized in patients who are aged 55 years or over.” There is a perception that melatonin production decreases with age, that the artificial rise in melatonin will simulate an early DLMO, and that the replenishment with exogenous melatonin will improve sleep efficiency (reduce awakenings) and total sleep time. It is for these reasons that melatonin has become popularized.

In the more than 30 years since the DLMO was defined, it is timely to reflect on its use and issues such as the variability across individuals, the effect of age and sex, how the melatonin is measured, and the DLMO is calculated and its relationship with chronotype. To achieve these aims, the mean saliva and plasma DLMO and ages of control/healthy participants were obtained from studies published after 1989. In addition, individual DLMO, MEQ, ages, and sex data were obtained directly from the authors of a subset of these publications published since 2010. Finally, to address the question of whether plasma melatonin levels decrease with age, the peak plasma melatonin data from a previous publication [33] was updated with data published since the original publication. The primary null hypotheses were that DLMO is not different across age groups and that DLMO and morningness/eveningness (MEQ) are not correlated. Secondary analyses investigated the effects on DLMO of the various analytical methods used to analyze saliva and plasma melatonin, and the methods used to calculate DLMO and sex differences. A reference range for DLMO versus age was constructed from the individual participant data. Finally, the nocturnal peak in plasma melatonin levels in normal participants from 20 to 80 years was analyzed.

## Methods

### DLMO versus age: mean data from published papers

A literature search of the PubMed and ScienceDirect databases was conducted using the terms “Melatonin onset,” “Dim light melatonin onset,” “Melatonin and plasma,” and “ Melatonin and saliva” from 1993. Papers that provided information on the sampling rate, analytical methods used, ages and numbers of participants, the method used to estimate DLMO, and the time of DLMO in clock time are used in this study. Additional papers were sourced through references in the papers and my personal database. Papers that did not report the DLMO in clock time, for example, used Z-scores, or calculated phase shifts of DLMO without reporting the actual values, etc., were excluded.

### Individual DLMO data obtained through data sharing and published papers

Email requests for the sharing of healthy control participant data were sent to the corresponding authors of 53 papers published between 2010 and 2021.

### Maximum plasma melatonin levels versus age: mean data from published papers

In a 1999 published study [33], data from 137 studies published between 1976 and 1997 with mean reporting plasma melatonin levels between 24:00 and 02:00 h and the ages of normal healthy adults were analyzed. A similar literature search covering the years 1997 to 2021 identified a further 41 studies for reanalysis.

### Statistics

For comparisons of DLMO across age groups using the data from the published studies, the means were weighted according to the number of participants in the study using IBM SPSS 28. Analysis of variance was used to test for differences between groups or ages. Post hoc analysis used Tukey’s HSD test. Differences with *p* < 0.05 were considered significant. For the linear regression analyses and preparation of graphs, GraphPad Prism 9 was used.

## Results

### DLMO versus age; mean data from published papers

**Figure 1.**
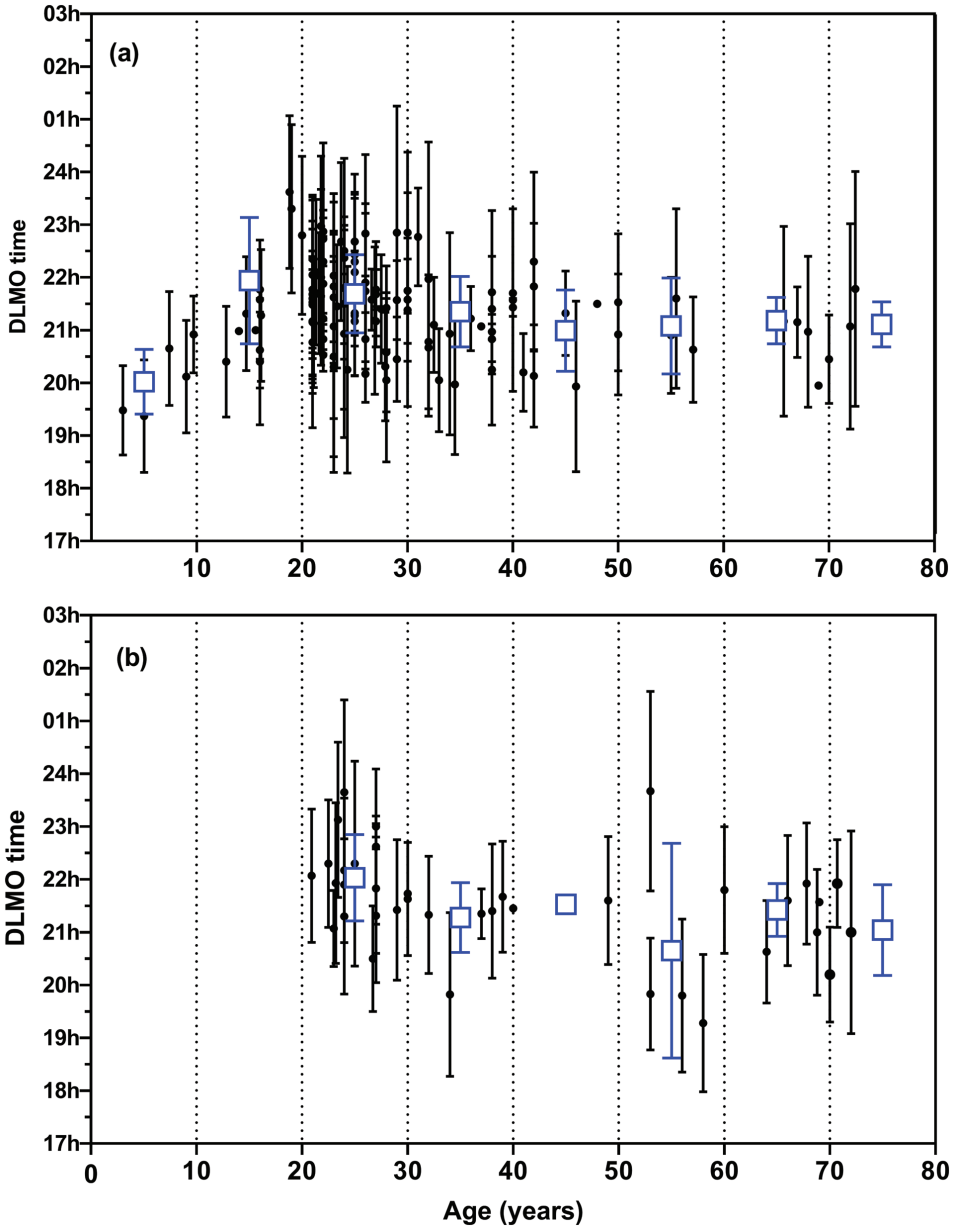
The saliva DLMO (A) and plasma DLMO (B) (mean ± SD) of groups of healthy participants from 121 published studies. The open squares are the n-weighted means ± SD of the 10-year age groups. Note that in some cases, no SD was available.

The searches identified 121 papers reporting saliva DLMO (Supplementary File 1) and 31 papers reporting plasma DLMO in healthy participants (Supplementary File 2). The DLMO values (with standard deviations, mean ages, and numbers of control/healthy participants) were sourced through the text, tables, or through digitization of graphs. Of these 121 publications, 8 also reported the DLMO in patients diagnosed with DSWPD. A further 6 papers found reported the DLMO only in patients with DSWPD and not controls. The entire saliva DLMO data set represented 3579 individuals, with the median number of participants in each group being 20 (range 4 to 170) and mean ages ranging from 3 to 73 years. The average standard deviation of the saliva DLMO data points were 1.2 h. For the plasma, DLMO studies the data set represented 818 participants, with the median number of participants in each group being 12 (range 3 to 104) and mean ages ranged from 21 to 72 years. The average standard deviation of the plasma DLMO data points were 1.2 h. Table 1 shows the weighted DLMO means (±SD) pooled into 10-year age groups. Figure 1 shows the published saliva and plasma DLMO (mean ± SD) plotted versus the mean ages of the groups. Saliva DLMO was earliest in children up to 10 years of age and the latest around 20 years of age. The saliva DLMO (weighted mean) for 20- to 29--year-old participants was 21.59 h (66 studies/1875 participants) and the plasma DLMO 22.1 h (17 studies/463 participants). The difference between the saliva DLMO of 20–29 and 70–79-year-old participants is 23 min and between 30–39 and 70–79-year-old participants is 10 min.

**Table 1.** The saliva and plasma DLMO from the literature across age groups

	0–9 years	10–19 years	20–29 years	30–39 years	40–49 years	50–59 years	60–69 years	70–79 years
Weighted mean saliva DLMO	20.02 ± 0.61 h	21.94 ± 1.20 h	21.60 ± 0.74 h	21.38 ± 0.68 h	20.97 ± 0.78 h	21.33 ± 0.46 h	20.96 ± 0.37 h	21.22 ± 0.40 h
Upper 95% CI	21.24 h	00.34 h	23.08 h	22.74 h	22.53 h	22.25 h	21.7 h	22.02 h
Lower 95% CI	18.80 h	19.54 h	20.12 h	20.02 h	19.41 h	20.41 h	20.22 h	20.42 h
# Studies	5	11	66	22	10	5	5	3
*n*	133	550	1875	450	252	133	82	104
Weighted mean plasma DLMO	—	—	22.15 ± 0.62 h	21.36 ± 0.57 h	21.54 ± 0.08 h	20.55 ± 1.64 h	21.43 ± 0.48 h	21.00 ± 0.43 h
# Studies	—	—	17	7	2	4	7	3
*n*	—	—	463	98	8	38	123	88

The data are the weighted means ± 1 SD for controls bundled into 10-year age groups based on the mean ages reported in the publications. Also shown are the upper and lower 95% confidence intervals. # studies indicate the number of publications used to generate the data. *n* = the total number of participants used in the publications.

### DLMO versus assay method and DLMO method; mean data from published papers

**Figure 2.**
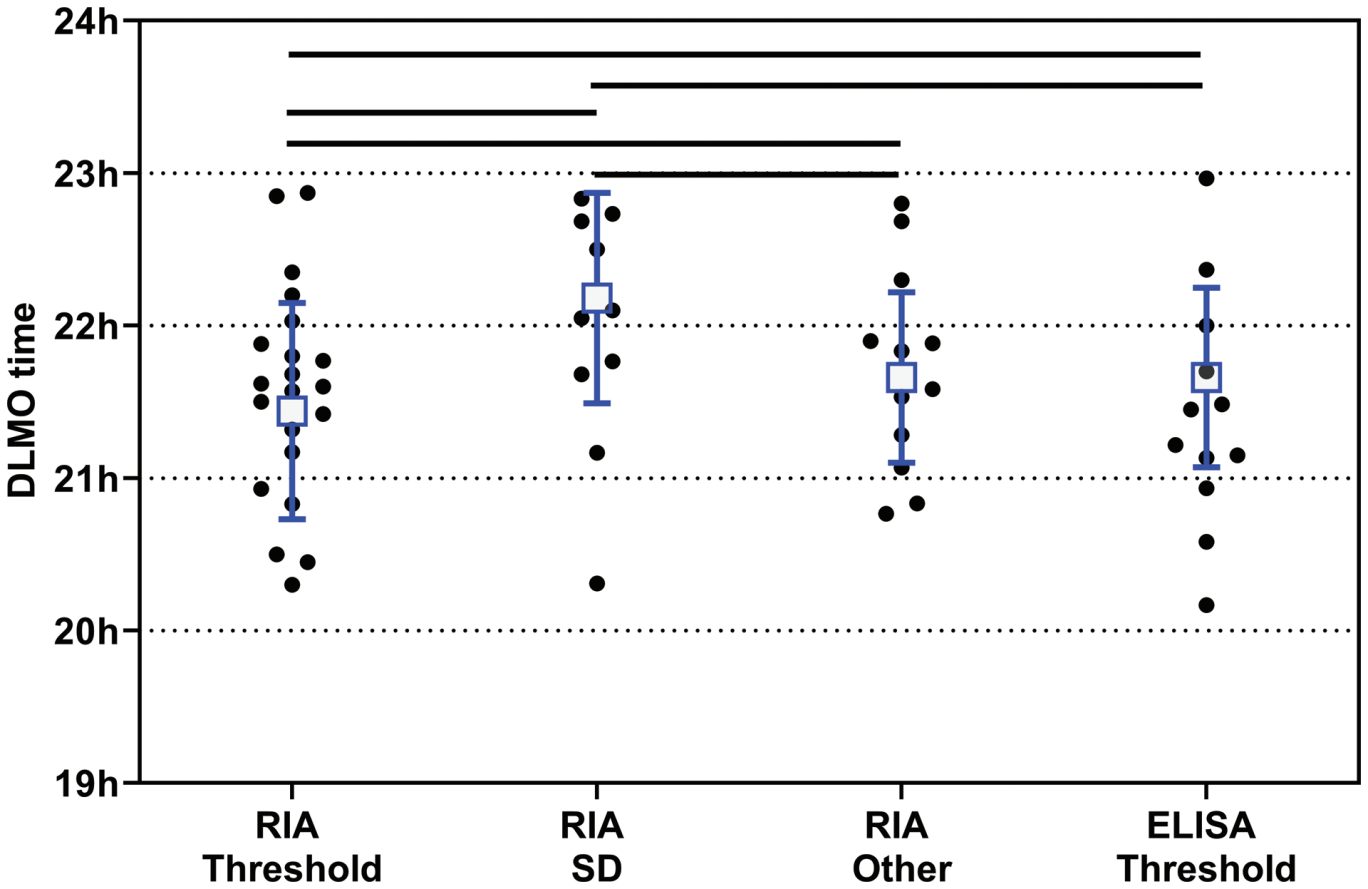
The published saliva DLMO means from healthy participants with mean ages between 20 and 29 years (the SD of the individual points were omitted for clarity). The squares and bars represent the n-weighted means ± SD for the following groups: (1) Melatonin assayed by RIA and DLMO determined as the time the melatonin concentration exceeded a threshold value, (2) melatonin assayed by RIA and the DLMO determined as the time it exceeded two standard deviations of the baseline, (3) melatonin assayed by RIA and the DLMO determined by other methods, and (4) melatonin assayed by ELISA and DLMO determined as the time the melatonin concentration exceeded a threshold value. The horizontal lines represent significant differences (*p* < 0.001) between the underlying groups. The data are from 21, 10, 12, and 12 studies, respectively.

To determine whether there are differences in the calculated saliva DLMO due to the melatonin assay methodologies used or the way the DLMO is calculated, the data from 64 studies conducted on healthy participants with mean ages between 20 and 29 years was analyzed. In the current study, the majority of saliva DLMO studies assayed melatonin by Radioimmunoassay (RIA) (72.4%), followed by 17.3% that used enzyme-linked immunosorbent assays (ELISA), 4.7% that used mass spectrometry, and 5.5% that used unspecified assays. The majority (78%) of RIAs were conducted with a Buhlmann/NovoLytiX direct saliva assay kit. For the saliva DLMO determined by ELISA, 54% used a Buhlmann/NovoLytiX kit, 23% use a IBL kit, and 18% used a Salimetrics kit. To compare the effects of assay technology on saliva DLMO, studies that included participants with mean ages in the most studied band (20–29 years) were assessed (Table 2). The proportions using each technology were similar to the main data set. The weighted mean saliva DLMO derived from studies that used RIA (21.66 ± 0.73 h; *n* = 1281) or ELISA (21.54 ± 0.71 h; *n* = 425) were comparable, whereas those studies using mass spectrometry (20.9 ± 0.38 h; *n* = 104) tended to report somewhat earlier DLMO. Note, however, that there were only four studies that utilized mass spectrometry assays. When the DLMO for this age group was calculated using an RIA or ELISA and the threshold beyond which the melatonin concentration must cross, (usually 3 pg/mL), or an RIA using the two SD of the baseline method, or other methods for determining DLMO (e.g. percentage of peak levels or the hockey stick method) there were significant differences in the DLMO (*p* < 0.001, Figure 2). The earliest published DLMO were obtained using the RIA/threshold method, followed by RIA/other methods and ELISA/threshold, with the RIA/SD method reporting the latest DLMO in this age group (Table 2). Only two papers used the standard deviation method with ELISA kits.

**Table 2.** The effects of assay method and DLMO calculation method on the DLMO for 20–29-year-old healthy participants from the literature

	RIA	ELISA	Mass Spec	Threshold	2 Std Dev	% of Peak	Other
DLMO	21.66 ± 0.73 h	21.54 ± 0.71 h	20.93 ± 0.38 h	21.53 ± 0.65 h	21.63 ± 0.88 h	21.88 ± 0.56 h	20.93 ± 0.61 h
# Studies	44	15	4	36	14	10	4
*n*	1281	425	104	1060	479	225	158

The data are the weighted means ± 1 SD for controls aged 20–29y. # studies indicate the number of publications used to generate the data. *n* = the total number of participants used in the publications.

### DLMO versus age; individual data from published papers

Of the 51 email requests for the sharing of individual data, 34 authors agreed to share their de-identified healthy control data from their 40 publications. Of a further five authors who initially agreed to share their data, three had failed to send any data up until the time of manuscript preparation and two provided data sets did not include ages of the participants or were not from healthy participants. Two authors indicated that they were not permitted to share data by their Ethics Committee or that the aims of the current study were too similar to their ongoing work. One further author was required by their university to obtain a nondisclosure agreement before data sharing and this was not finalized before manuscript preparation. Finally, 10 corresponding and coauthors failed to respond to multiple attempts to contact them about the data sharing. A full set of DLMO, age, and MEQ data was available for 26 papers, with DLMO and age only, available for a further 14 papers (Supplementary File 3). Literature searches identified a further seven papers that published individual age and DLMO data in tables or graphs in the main manuscript or in Supplementary files and six papers that published individual DLMO and MEQ values in tables or graphs. A total of 53 papers provided data for the final analyses [34–86].

**Figure 3.**
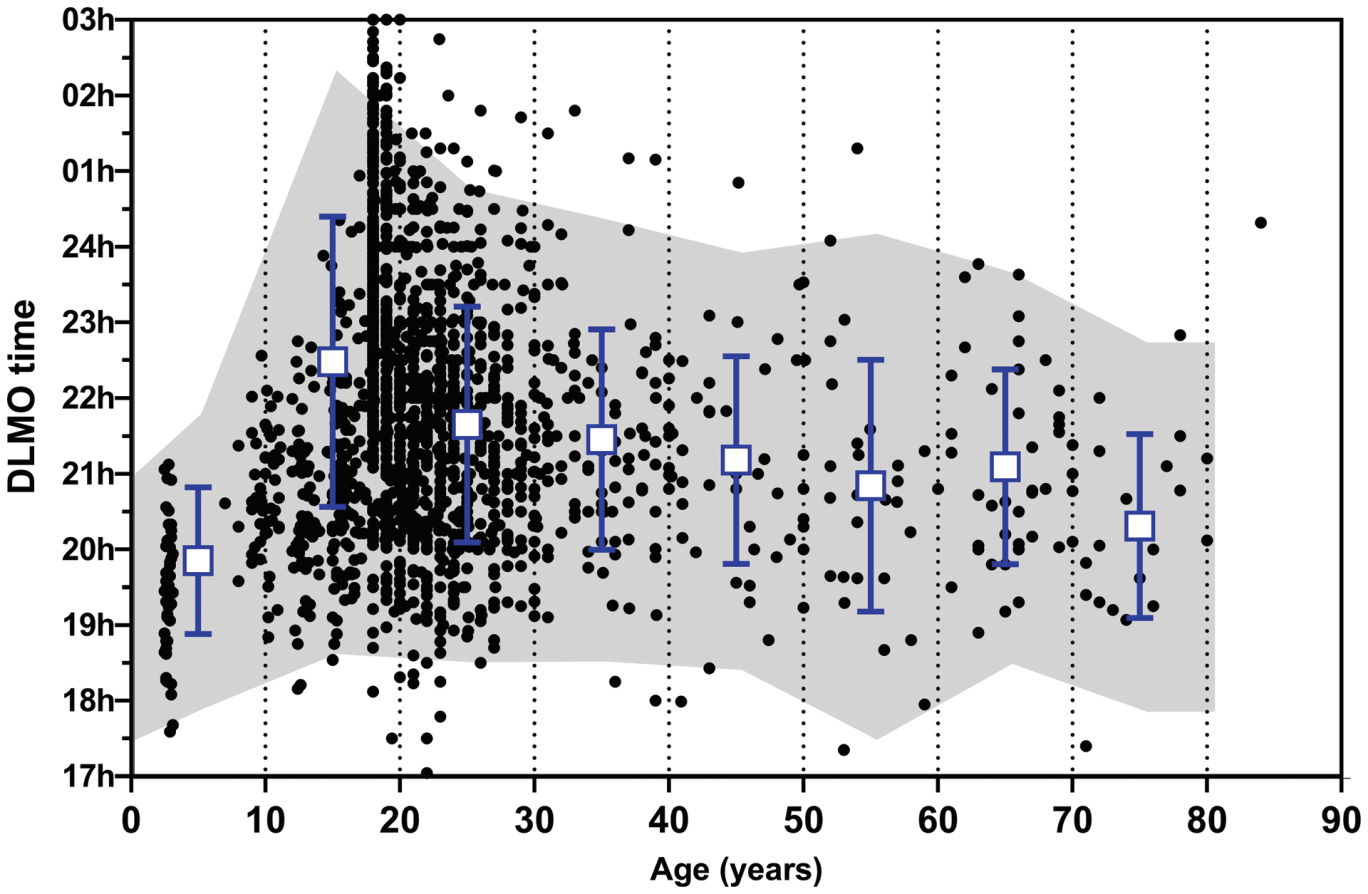
Individual saliva DLMO for 1721 healthy participants, plotted versus their ages. The shaded area represents two SD of the n-weighted means for each 10-year age category. The squares and bars are the means ± SD.

To determine whether the saliva DLMO changes with age, the DLMO from 1749 participants were plotted against their ages (Figure 3). The mean ± SD for 10-year age groups (0–9 years, 10–19 years, etc) was calculated (Table 3). Between early childhood and late adolescence, the saliva DLMO was delayed by approximately 2 h 38 min. From around 25 years of age to 75 years of age, however, the DLMO gradually advanced by approximately 1 h 20 min. Analysis of variance comparing the 10-year age bands from 0–9 to 70–79 years revealed a significant between groups (*p* < 0.001). The 80–89 year group was omitted from the analysis because of a very small number of participants. Post hoc analysis (Tukey HSD) indicated the DLMO for 0–9 year-olds was earlier than the 10–19, 20–29, 30–39, 40–49, and 60–69-year-old groups (*p* < 0.004). The DLMO for the 10–19 year-olds was later than all the other age groups (*p* < 0.001). The DLMO for the 20- to 29-year-old group was later than the 70–79 year olds (*p* = 0.008). As was also evident from the published means reported in Figure 1, the saliva DLMO is extremely variable within age cohorts. Using the DLMO ± 2 standard deviations, a reference range was constructed for the 10-year age cohorts (Table 3) and also shown as the shaded area in Figure 3.

**Table 3.** The saliva DLMO across age groups using individual data sets from the literature

	0–9 years	10–19 years	20–29 years	30–39 years	40–49 years	50–59 years	60–69 years	70–79 years	80–89 years
DLMO (h)	19.85 ± 0.97 h	22.48 ± 1.92 h	21.65 ± 1.56 h	21.45 ± 1.46 h	21.18 ± 1.37 h	20.84 ± 1.67 h	21.09 ± 1.29 h	20.31 ± 1.22 h	21.88 ± 2.18 h
Upper 95% CI	21.79 h	02.32 h	00.77 h	00.37 h	23.92 h	24.18 h	23.67 h	22.75 h	02.24 h
Lower 95% CI	17.91 h	18.64 h	18.53 h	18.53 h	18.44 h	17.50 h	18.51 h	17.87 h	17.52 h
*n*	75	713	698	117	44	36	42	21	3

The data are the means ± 1 SD for controls bundled into 10-year age groups from the shared data sets. Also shown are the upper and lower 95% confidence intervals. *n* = the total number of participants in each age bracket. Analysis of variance comparing the 10-year age bands from 0–9 to 70–79 years revealed a significant between groups difference (*p* < 0.001). The 80–89 year group was omitted from the analysis because of a very small number of participants. Post hoc analysis (Tukey HSD) indicated the DLMO for 0–9 year-olds was earlier than the 10–19, 20–29, 30–39, 40–49, and 60–69-year-old groups (*p* < 0.004). The DLMO for the 10–19 year-olds was later than all the other age groups (*p* < 0.001). The DLMO for the 20–29-year-old group was later than the 70–79 year-olds (*p* = 0.008).

Within the dataset of published papers, there were 11 papers (three saliva DLMOs and eight plasma DLMO) that directly compared the DLMO of young (<30 years) versus older healthy participants (>50 years) (Supplementary File 4). Ten out of the 11 papers reported earlier DLMO in the older participants compared with the young.

### Effects of sex on DLMO across age groups; individual data from published papers

Using the individual DLMO data set, the effect of sex on DLMO was evaluated across the age groups. There was no effect of sex on DLMO (Table 4).

**Table 4.** The effects of sex on DLMO across age groups

Age band (years)	0–9	10–19	20–29	30–39	40–49	50–59	60–69	70–79
Female DLMO	20.94 ± 0.81	22.86 ± 1.75	21.71 ± 1.46	21.65 ± 1.03	21.08 ± 1.24	21.63 ± 2.22	21.09 ± 1.35	20.05 ± 0.85
Female *n* =	7	315	279	26	12	11	13	4
Male DLMO	20.54 ± 0.46	23.08 ± 1.98	21.91 ± 2.34	21.64 ± 2.05	22.01 ± 0.59	20.92 ± 2.26	21.42 ± 1.29	20.92 ± 1.22
Male *n* =	8	244	195	17	8	10	11	7
Female MEQ	42.50 ± 5.09	46.38 ± 9.28	49.48 ± 9.88	54.11 ± 11.49	56.33 ± 10.84	56.64 ± 11.68	58.31 ± 8.82	61.75 ± 9.91
Female *n* =	6	290	277	27	12	11	13	4
Male MEQ	39.75 ± 4.33	43.74 ± 9.06	48.73 ± 9.81	52.50 ± 10.05	55.06 ± 7.65	59.40 ± 7.57	59.55 ± 8.71	61.43 ± 12.23
Male *n* =	8	232	192	16	8	10	11	7
Combined MEQ	40.93 ± 4.70	45.20 ± 9.24	49.18 ± 9.85	53.51 ± 10.88	55.83 ± 9.49	57.95 ± 9.80	58.88 ± 8.60	61.55 ± 10.92

The data are the means ± 1 SD for control males and females bundled into 10-year age groups from the shared data sets. *n* = the number of participants in each age band. ANOVA indicated a significant effect of age on DLMO (*p* < 0.001), but no sex effects or interaction. Similarly, there was a significant effect of age on MEQ (*p* < 0.001), but no sex effects or interaction.

### DLMO in patient groups diagnosed with DSWPD versus age

**Figure 4.**
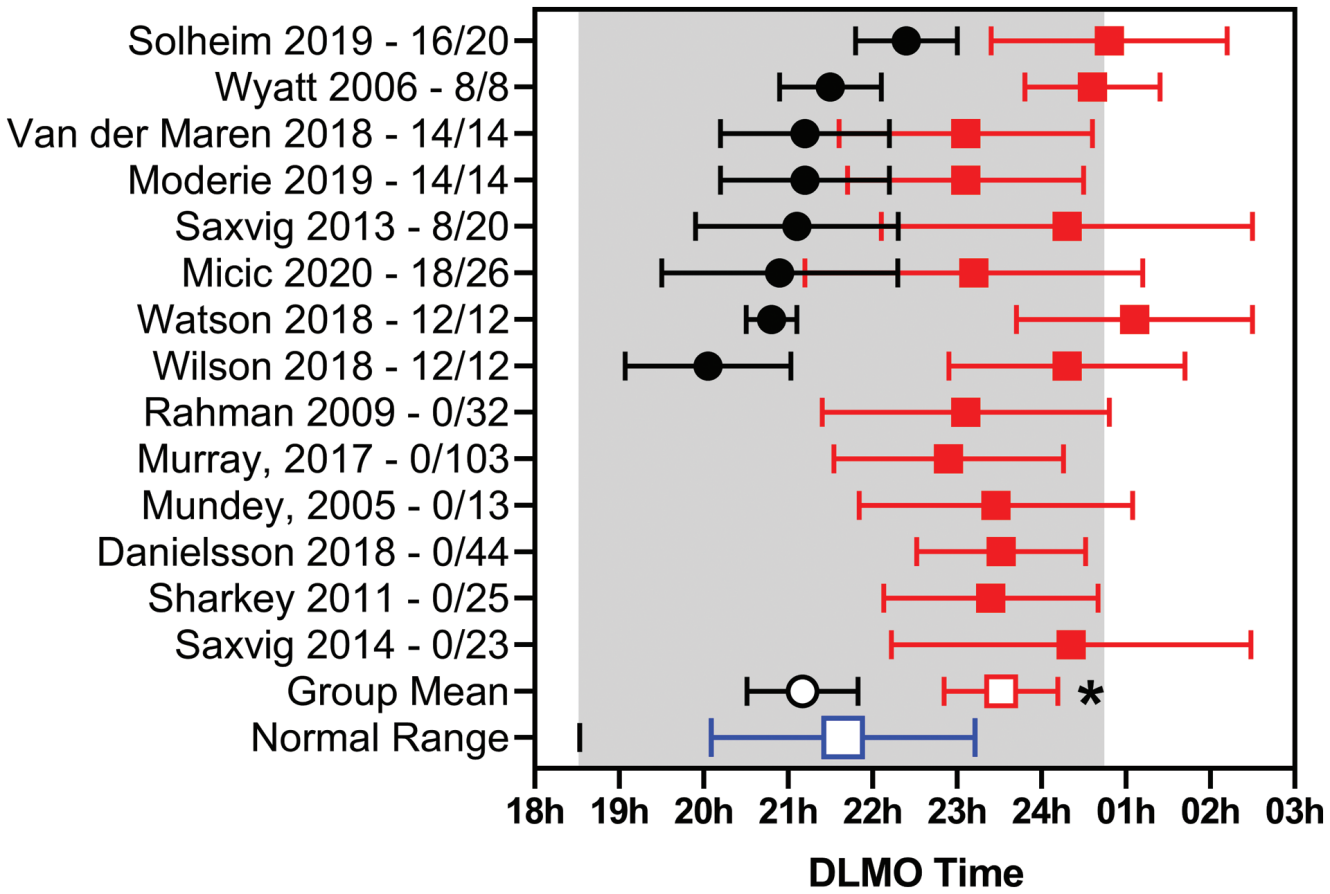
The published saliva DLMO (mean ± SD) for healthy control participants (filled circles) and patients diagnosed with DSWPD (filled squares) aged between 20 and 31 years. The author and publication years are shown, together with the number of participants studied. Group n-weighted means ± SD for the healthy controls (open circle) and DSWPD (small square) are shown. Also shown is the n-weighted mean ± SD (large square) for this age group. The shaded area represents two SD of the means of the individual data.

DSWPD has long been associated with a later DLMO and so it was of interest to compare the published results with the current reference ranges. A large majority of the studies investigated participants with mean ages between 20 and 31 years. Figure 4 shows the DLMO for 14 studies (13 saliva, 1 plasma), 8 of which included healthy controls in this age group. The weighted mean DLMO for the healthy controls was 21.17 h ± 0.64 h (*n* = 101) compared with 23.97 ± 0.75 h (*n* = 124) for the associated participants living with DSWPD in those eight studies. In each case, the authors reported that the DLMO was significantly later for participants with DSWPD. Note, however, that the mean DLMO was within the reference range for 12 of the 14 studies. For all 14 DSWPD studies, the weighted mean DLMO was 23.50 ± 0.67 (*n* = 345). The differences between the weighted means for the controls and DSWPD groups were significant (*p* < 0.001). Note that two studies applied additional selection criteria for their DSWPD participants; one [23] used sleep diary and PSG sleep latency results to differentiate participants while the other [27] used a “normal” DLMO to exclude participants from the DSWPD diagnosis. The DLMO from these 14 DSWPD studies and a further 6 DSWPD studies whose participants' ages were outside the range used above are shown in Supplementary File 5 together with their references and criteria used to diagnose DSWPD.

### Nocturnal peak melatonin levels in healthy participants across age groups; mean data from published papers

**Figure 5.**
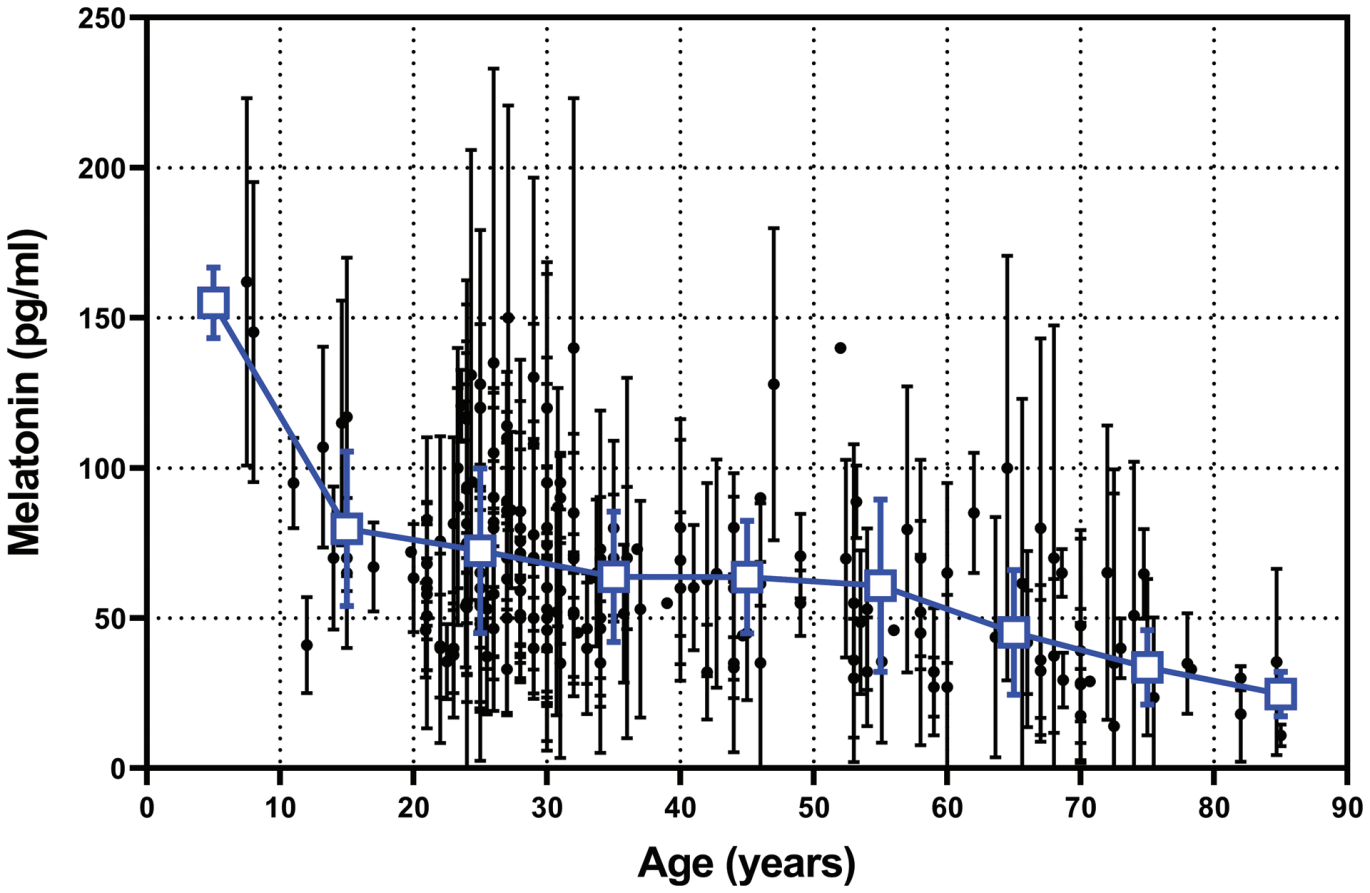
The published peak (24 h–03 h) plasma melatonin levels (mean ± SD) in healthy participants from 179 studies versus age. The n-weighted means ± SD are also shown (squares).

Along with potential changes in the onset of melatonin with age, it is of interest whether peak melatonin secretion also changes with age. Figure 5 shows an updated version of a graph published by Kennaway et al. [33] of mean peak plasma melatonin concentration from early childhood into old age which includes 41 studies published since then. See Supplementary File 6 for references. Plasma melatonin is highest prior to late adolescence. From the mid-20s until the mid-50s, there is a gradual decline in peak levels from 72 to 61 pg/mL. Thereafter, peak melatonin levels appear to decline at a faster rate to be approximately 25 pg/mL in participants in their mid-80s. As can be seen in Table 5, however, the variability within and between studies is extremely high (the average SD is 23 pg/mL).

**Table 5.** Weighted means of peak melatonin levels (pg/mL) across ages from the literature

	0–9 years	10–19 years	20–29 years	30–39 years	40–49 years	50–59 years	60–69 years	70–79 years	80–89 years
Melatonin (pg/ml)	155	79.7 ± 25. 8	72.4 ± 27.4	63.8 ± 21.7	63.6 ± 18.7	60.8 ± 28.7	45.2 ± 20.8	33.6 ± 12.4	24.8 ± 7.4
# Studies	2	10	78	42	19	17	15	16	4
(*n*)	24	118	910	531	250	214	293	362	74

The data are the weighted means ± SD of peak (2400 hours–0300 hours) melatonin levels derived from the literature. # Studies indicate the number of age groups within the 10-year age bands. *n* = the cumulative number of participants in each age band.

Supplementary File 6 shows data from 18 published studies in which peak plasma melatonin was measured in healthy participants in two different age cohorts (“young” vs. “old”) under the same conditions. In the table, the data are stratified by the change in peak melatonin per year between the age groups. The range of results is very large. The median of the 18 studies was 0.51 pg/mL per year with 9 studies reporting changes <0.5 pg/mL/year with a mean change of 0.26 pg/mL/year. The remaining nine studies had a mean change of 1.04 pg/mL/year, almost four times the rate. In those studies reporting a rapid decline in melatonin levels, the *n*-weighted mean of the “young” group was significantly higher than the group that had the slowest decline.

### Relationship between DLMO and morningness/eveningness; individual data from published papers

**Figure 6.**
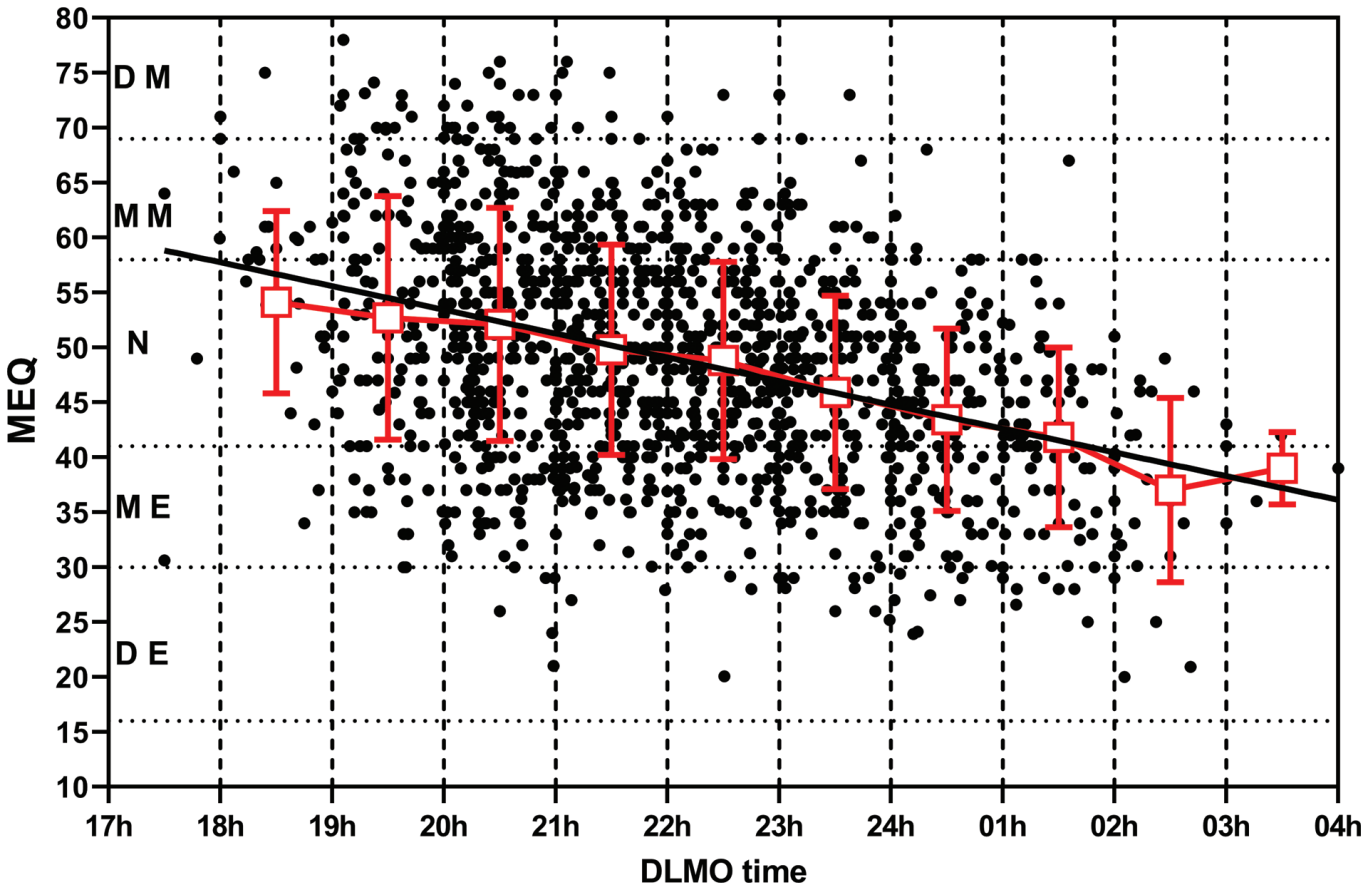
Individual MEQ scores were plotted versus the saliva DLMO for healthy participants not corrected for age. The MEQ (mean ± SD) for each 1-hour DLMO block is plotted (squares) together with the result of the simple linear regression line of the full data set. DE, definite evening; ME, moderate evening; N, neither morning nor evening; MM, moderate morning; DM, definite morning.

Using the shared individual data and data obtained from publications, it was possible to determine the relationship between the MEQ and saliva DLMO (Figure 6). There was a significant correlation (*p* < 0.0001) between the two measures with high MEQ scores (morningness) associated with an early saliva DLMO and lower MEQ scores (eveningness) associated with a late DLMO. Of the 29 data sets used for this analysis, individual regression analysis indicated a significant deviation from a zero slope in 15 and not significant in 14 sets (Supplementary File 7). This is likely to be a result of the extreme variation in MEQ and DLMO between participants.

### Relationship between age and morningness/eveningness; individual data from published papers

**Figure 7.**
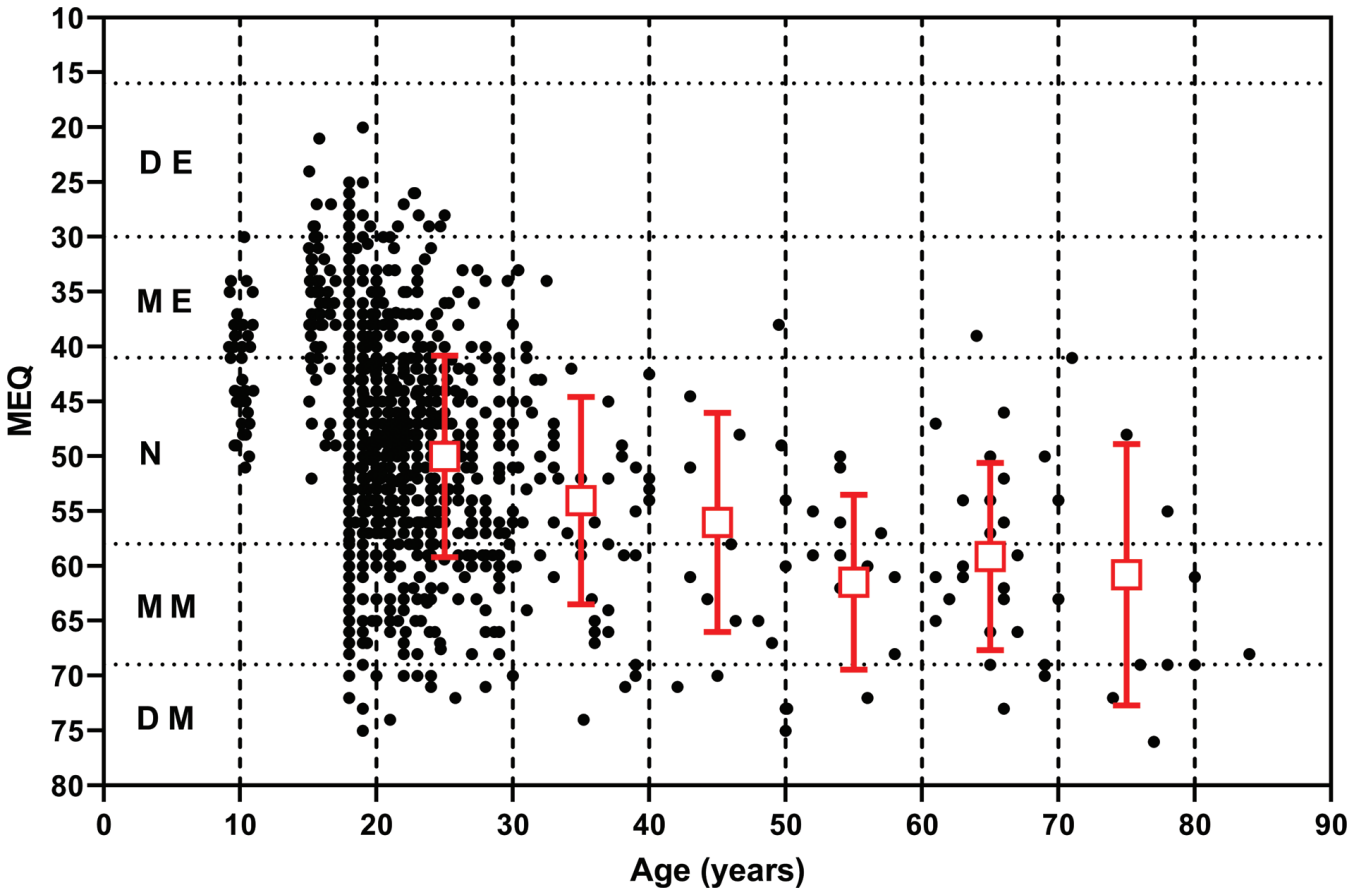
Individual MEQ scores were plotted against age for healthy participants. The MEQ (mean ± SD) for each 10-year age group between 20 and 80 years is also plotted (squares). DE, definite evening; ME, moderate evening; N, neither morning nor evening; MM, moderate morning; DM, definite morning.

Using the shared and published individual data sets, it was possible to assess the impact of age on MEQ scores. Figure 7 shows that, with increasing age, there is a change from a tendency toward eveningness in younger participants to morningness in older participants. There was no effect of sex on the MEQ across the age ranges (Table 4).

### DLMO, MEQ, bedtime, and sleep onset time across age

**Figure 8.**
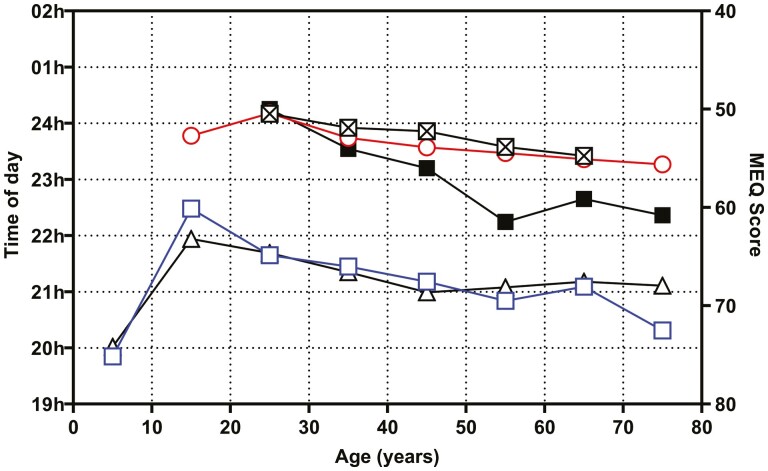
Summary of the saliva DLMO and MEQ scores from the current study and mean bedtimes and mean sleep onset from the literature. The n-weighted mean saliva DLMO from published studies (triangles). Mean saliva DLMO from the individual values in the current study (open squares). The mean MEQ scores from the individual values in the current study (filled squares). The mean bedtimes were redrawn from the paper by [88]. The mean sleep onset was redrawn from the paper by [87].

In Figure 8, the weighted means of the published mean DLMO, the individual DLMO means and the MEQ from this study are plotted together with the mean bedtimes and mean sleep onsets from two independent studies [87, 88]. The main points to be made from this summary graph are that (1) DLMO is later in late adolescence and slowly advances with age, (2) in late adolescence eveningness gives way to a tendency to morningness with age, and (3) the time of going to bed and getting to sleep occurs earlier as people age.

## Discussion

The primary null hypotheses of this study were that DLMO is not different across age groups and that DLMO and morningness/eveningness (MEQ) are not correlated. Secondary analyses investigated the effects of the various analytical methods used to analyze saliva and plasma melatonin on DLMO, the methods used to calculate it, and the effect of sex. Finally, changes in the nocturnal peak in plasma melatonin levels in normal participants from 20 to 80 years were analyzed to determine whether production decreases with age.

To address the first question, a search of the literature was conducted and mean saliva and plasma DLMO data for groups of healthy participants was obtained and plotted against the mean ages of the groups. The saliva DLMO was earliest in children under 10 years of age and highest in late adolescence. After this age, there was a gradual advance of the saliva DLMO. Although there were fewer studies reporting plasma DLMO, the trend toward a slightly earlier DLMO in younger people was evident. Following a request to more than 50 authors for their de-identified individual DLMO, age, MEQ scores, and sex data for healthy participants, a similar analysis was conducted on DLMO/age pairs. This was expected to confirm the results of the primary analysis of the published means, but provide a more reliable estimate of the variability of the saliva DLMO. The latest DLMO was associated with late adolescence and slowly advanced with age up to the mid-70s. There was an extraordinary degree of variability within the age cohorts such that the putative reference range (based upon 2 standard deviations of the mean) for the 10-year age cohorts was very large

The results of this part of the study are of particular importance since in a highly cited review published in 2018 it was stated that “Older subjects show *an increased lag from sunset to the onset of melatonin pulse* and to the melatonin pulse peak and between melatonin secretion peak and the middle of the sleep period.” [89] No references were used to support the statement. If true, a delay in DLMO with age might be used to justify the use of exogenous melatonin to correct the perceived abnormality in melatonin timing. In support of the current study, a literature search identified 11 papers (3 saliva DLMO and 8 plasma DLMO) that compared DLMO in young versus older healthy participants under the same conditions, with 10 of the papers reporting that the DLMO was indeed *earlier* in the older participants.

Although the time of onset of melatonin production provides a quantifiable estimate of circadian phase, questionnaire-based methods have been developed to assess what is termed morningness and eveningness. The Horne and Ostberg questionnaire (MEQ) has been used in many studies and has been validated in several languages while other similar tests were developed later [90, 91]. A shorter form of the questionnaire was developed by Smith et al. in an attempt to address perceived deficiencies of the MEQ [92]. More recently, a new questionnaire has been developed, the Munich ChronoType Questionnaire (MCTQ) which addresses more specifically the *actual* times of sleep during weekdays and weekends [93]. The mid-sleep on free days is highly correlated with the MEQ and shows that young children are generally earlier chronotypes, progressively becoming later until the age of 20 years and then earlier again with age [94]. The MEQ in the current study was found to change from moderate eveningness to moderate morningness with age as previously reported [95, 96]. It is beyond the scope of this paper to discuss the various advantages of the questionnaires, but it is clear that the MEQ is older and has been more widely used than the MCTQ and this is reflected by few papers reporting simultaneous DLMO and MCTQ results.

In the current study, the saliva DLMO was significantly correlated with the MEQ in healthy participants. Previous small studies using healthy participants [40, 85] or a mixture of healthy participants and participants diagnosed with DSWPD [82] found strong associations between MEQ and DLMO and midpoint of sleep on work–free days, sleep-corrected and DLMO. Although the correlation reported in the current study is interesting, the high degree of variability in both measures strongly suggests that the MEQ should not be used as a surrogate/proxy measure for DLMO.

The two major null hypotheses in this very large study are rejected; DLMO clearly advances with age after late adolescence and DLMO and MEQ are highly correlated.

When assessing DLMO in the laboratory, there are a number of methodological issues that need to be considered; what assay to use for the melatonin measurement an what objective method to use to determine when melatonin secretion has started. With respect to the first point, I have written several reviews recently about melatonin assays in which I have criticized many of the ELISA that have appeared on the market in the last 10–15 years [6–10, 97, 98]. Poor assay design, specificity, and sensitivity have meant that many are incapable of reporting valid daytime melatonin levels in saliva or plasma. We know that melatonin is not actively synthesized during the daytime and therefore that plasma melatonin levels are expected to be less than 4 pg/mL based on mass spectrometry and less than that for saliva. It is clear that to accurately determine saliva DLMO, RIA, and ELISA kits from Buhlmann/NovoLytiX and IBL and ELISA kits from Salimetrics are to be preferred over other kits. Note, however, that the ELISA kits from small companies can still cause problems in saliva [98], especially when used for direct (nonextracted) assay of plasma melatonin [6–10, 97, 98].

The other issue that can affect the DLMO is the calculation method used. The original plasma DLMO was defined as the time that the plot of melatonin concentration versus time of day crossed 10 pg/mL [11]. A mass spectrometry assay was used which had a very high sensitivity (1 pg/mL) and rarely reported values before sleep that exceed this concentration. With the understanding that saliva melatonin levels are approximately one-third of those of plasma, a comparable threshold level would be around 3–4 pg/mL [20]. This low threshold level for saliva melatonin measurement produces a technical challenge because not all assay kits are capable of measuring/reporting the expected daytime levels of <1 pg/mL. In part for this reason other methods have been used. First, a statistical method based on levels exceeding 2 standard deviations of the mean baseline concentrations [12]. This is used when an assay either lacks sensitivity or specificity or detects daytime levels in excess of 3–4 pg/mL because of nonspecific interference. Another method is to determine the time of day that the melatonin concentration reaches a certain percentage (often 25%) of the peak melatonin level. This is more commonly used for plasma DLMO when samples are collected throughout the night, as opposed to saliva collections that usually cease around midnight. Yet another method used infrequently is the “Hockey stick method” [99]. Across studies on 20 to 29 year olds, the choice of DLMO calculation method had little impact on the DLMO. Given the variability between studies and within studies, it seems clear that the use of either a low threshold or a standard deviation of baseline levels approach may both be acceptable.

With respect to the effect of sex on DLMO, the current study has shown no difference across the age groups. In addition, we failed to detect any sex differences in the MEQ score across the ages. This is in contrast to the results of a large study using the MCTQ [94] which found that males had a later chronotype (eveningness) than women up until 50 years of age. This difference is likely to be a reflection of the different questions across the questionnaires especially those related directly to sleep times.

DLMO is recognized as a useful circadian marker since it is dependent on the suprachiasmatic nucleus timing system. It can be influenced by light exposure, such that light presented in the evening can phase delay the DLMO, while light in the early morning can advance the DLMO [100]. Furthermore, melatonin administration can also alter the timing of the DLMO, such that administration in the evening promotes an advance of the DLMO and morning administration can delay the DLMO [101]. DSWPD is diagnosed clinically after a careful evaluation of the patient’s sleep history using International Classification of Sleep Disorders (ICSD-3) criteria. Several authors have suggested that the DLMO should be used as well [23, 24], but the technique has not been widely accepted or used. It would appear that not all patients with DSWPD actually have a delay in their circadian timing system. In a recent study [27], Murray et al., for example, found that 43% of clinically diagnosed participants with DSWPD could be classified as having non-circadian DSWPD (defined as having a DLMO time greater than 30 min before their desired bedtime). In the current study, 12 out of the 14 DSWPD studies reviewed had the mean DLMO within the normal range. When compared with healthy controls, the mean DLMO of the participants with DSWPD was clearly and significantly later, but the large variance is suggestive of many of those diagnosed with DSWPD having normal melatonin timing. Measurement of DLMO in DSWPD may, however, have an important role in selecting patients who have DLMO outside the reference range for melatonin therapy [102].

The final part of the current study revisited the question of whether the production of melatonin decreases with age. If the production of melatonin decreases dramatically with age, it could impact on the determination of the DLMO, especially when a fixed threshold of 3 pg/mL is used. It has been repeatedly reported that melatonin production decreases with age, with the inference that people older than 50 years are melatonin deficient. In a previous analysis [33], this conclusion was challenged following a re-examination of published data and the author’s own research. The conclusion from that study was that “melatonin production is lower in older people, but the change occurs very early in life, around 20-30 years of age.” In the current study, peak plasma melatonin data from the original paper have been supplemented with results published since 1998 and a similar conclusion can be drawn. It is not until 65 years and older that peak melatonin production dips below 50 pg/mL. More importantly, like other aspects of melatonin rhythmicity, there is a very high level of variability between individuals and between primary studies. Two recent systematic reviews on melatonin production in healthy older participants [103, 104] showed unequivocally that they had melatonin rhythms of high amplitude. The current study of DLMO across age groups has shown that the DLMO actually occurs earlier in older participants, which is not to be expected in older people who are somehow melatonin deficient.

The (false) premise that a large proportion of older people is “low-secretors” or “melatonin deficient,” together with the observation that older people often with primary insomnia, has been seized upon as a reason to recommend the prescription of melatonin either as a pharmaceutical product or as a “supplement.” Is there in fact a link between low melatonin secretion and insomnia? There have been at least 10 papers published that concluded that there is no significant relationship between the *amount* of melatonin produced at night and insomnia. These 10 papers [105–114] have been cited a total of 376 times (on average two citations a year since publication), while three papers [115–117] that did report a relationship have been cited a total of 732 times (on average 9.6 times per year since publication (Supplementary File 8). Since the elderly are already producing significant amounts of melatonin [103, 104], the amount of melatonin is not related to insomnia and the DLMO actually occurs *earlier* than in younger people, we might question the justification for melatonin administration as a “replacement.” Of course, exogenous melatonin may still be beneficial for helping insomniacs achieve better sleep quality, increase total sleep and accelerate sleep onset, but a recent review of its efficacy would indicate that these effects are modest [32].

This study has some limitations, but they are unlikely to affect the conclusions reached. This was not a formal systematic review, but the searches conducted by the author uncovered more than 150 relevant publications to produce the main data set. These papers came from laboratories in 15 different countries and used different methodologies. It was considered unreasonable to expect raw data to be available for analysis from all the papers and an arbitrary cutoff date of 2010 was decided upon for this aspect of the study. Approximately 80% of the authors contacted by the author responded which is an excellent strike rate. It was unfortunate that 10 authors could not be contacted or refused to reply to the approaches as their data would have enriched the study. Most authors did not consider the supply of de-identified data from healthy participants raised any particular ethical concerns; indeed many journals now expect authors to provide original data sets upon reasonable request, while others encourage the publishing of complete data sets in Supplementary Files.

In a study such as the current one, it is not possible to control for the potential confounders of light exposure (i.e. how dim was the light?), possible changes in posture between sampling, or adherence to the designated sampling times when samples were collected at home. The selection of the healthy control participants may also have had a minor influence on the measured outcomes if there was an exclusion of extreme chronotypes from the experiments. For example, one study stated that “healthy controls were deemed eligible for further study if they had a DLMO time of earlier than 22.00 h” [118]. Another limitation of the current study which could not be avoided is the extraordinary age bias of the studies. More than 50% of the published studies included here were conducted on participants whose ages ranged from 20 to 29 years, while only 11% investigated the saliva DLMO on participants aged over 50 years. This is despite the fact that this age group is targeted by the vitamin and pharmaceutical industries because of the perception that this group has a high incidence of sleep disorders. Finally, there is a small proportion of the normal population who can be considered “low melatonin secretors” for whom the DLMO cannot be determined by any of the standard methods [63, 119, 120]. The incidence of these low secretors is difficult to establish due to different assay methodologies and sensitivities but is probably less than 10%.

In summary, the current study of published saliva and plasma DLMO in healthy control participants has found that the DLMO is extremely variable within age groups and within measures of morningness/eveningness. The basis of the variability is not known. One possible contributor is the variability in the intrinsic circadian period between participants with those having longer periods having a later DLMO. Against this notion, however, is the fact that the endogenous period of humans has been reported to be 24.15 h, with an SD of approximately 12 min as opposed to the approximately 90 min SD of DLMO reported here [4]. Other possible causes are variations in light exposure and wake times, which could not be controlled for in the current study. The time of the DLMO advances with age after early adulthood and is correlated with the MEQ score such that those with moderate eveningness have a later DLMO. A reference range was calculated for 10-year age bands that could be useful as a guide for clinicians investigating sleep timing disorders. For example, it was found that large proportion of patients clinically diagnosed with DSWPD had saliva DLMO within the normal reference range implying that they may not have had an underlying circadian timing disorder, but that there were other causes of their delayed sleep patterns. Finally, while peak nocturnal melatonin levels in healthy older people decrease slowly with age after early adulthood, those aged ≥70 years still maintain high amplitude melatonin rhythms.

## Supplementary Material

zsad033_suppl_Supplementary_File_1

## Data Availability

The individual data underlying this article were provided by multiple third parties and further sharing may require their permission. Data from the published studies analyzed here will be shared on reasonable request to the corresponding author.
