# Supplementary material for: The dim light melatonin onset across ages, methodologies, and sex and its relationship with morningness/eveningness
Source: Sleep. 2023 Feb 17;46(5):zsad033. doi: 10.1093/sleep/zsad033 (PMC10171641; doi:10.1093/sleep/zsad033)
Supplement: zsad033_suppl_Supplementary_File_1 [file zsad033_suppl_supplementary_file_1.pdf]

The Dim Light Melatonin Onset (DLMO) across ages, methodologies and sex and its relationship with Morningness/Eveningness.

David J. Kennaway

Robinson Research Institute and Adelaide School of Medicine,

University of Adelaide,

Adelaide Health and Medical Science Building,

North Terrace, Adelaide,

South Australia, Australia.

Corresponding author:

David J. Kennaway

Email: david.kennaway@adelaide.edu.au

https://orcid.org/0000-0002-5864-3514

Supplementary File 1

The papers reporting Saliva DMLO used in the current study

[1] LeBourgeois MK, Carskadon MA, Akacem LD, Simpkin CT, Wright KP, Achermann P, et al. Circadian phase and its relationship to nighttime sleep in toddlers. J Biol Rhythms. 2013;28:322-31.

[2] Akacem LD, Wright KP, Jr., LeBourgeois MK. Bedtime and evening light exposure influence circadian timing in preschool-age children: A field study. Neurobiol Sleep Circ Rhythms. 2016;1:27-31.

[3] Moreno JP, Hannay KM, Walch O, Dadabhoy H, Christian J, Puyau M, et al. Estimating circadian phase in elementary school children: Leveraging advances in physiologically-informed models of circadian entrainment and wearable devices. Sleep. 2022.

[4] Van Der Heijden KB, Smits MG, Van Someren EJW, Boudewijn Gunning W. Prediction of melatonin efficacy by pretreatment dim light melatonin onset in children with idiopathic chronic sleep onset insomnia. J Sleep Res. 2005;14:187-94.

[5] Higuchi S, Lee SI, Kozaki T, Harada T, Tanaka I. Late circadian phase in adults and children is correlated with use of high color temperature light at home at night. Chronobiol Int. 2016;33:448-52.

[6] Stone JE, Phillips AJK, Chachos E, Hand AJ, Lu S, Carskadon MA, et al. In-person vs. home schooling during the COVID-19 pandemic: Differences in sleep, circadian timing, and mood in early adolescence. J Pineal Res. 2021;71:e12757.

[7] Herz RS, Van Reen E, Barker DH, Hilditch CJ, Bartz AL, Carskadon MA. The influence of circadian yiming on olfactory sensitivity. Chem Senses. 2017;43:45-51.

[8] Dolsen MR, Wyatt JK, Harvey AG. Sleep, circadian rhythms, and risk across health domains in adolescents with an evening circadian preference. J Clin Child Adolesc Psychol. 2019;48:480-90.

[9] Goldman SE, Alder ML, Burgess HJ, Corbett BA, Hundley R, Wofford D, et al. Characterizing sleep in adolescents and adults with autism spectrum disorders. J Autism Dev Disord. 2017;47:1682-169.

[10] Misiunaite I, Eastman CI, Crowley SJ. Circadian phase advances in response to weekend morning light in adolescents with short sleep and late bedtimes on school nights. Front Neurosci. 2020;14:99.

[11] Reichert CF, Veitz S, Bühler M, Gruber G, Deuring G, Rehm SS, et al. Wide awake at bedtime? Effects of caffeine on sleep and circadian timing in male adolescents - a randomized crossover trial. Biochem Pharmacol. 2020;191:114283.

[12] Agostini A, Carskadon MA, Dorrian J, Coussens S, Short MA. An experimental study of adolescent sleep restriction during a simulated school week: changes in phase, sleep staging, performance and sleepiness. J Sleep Res. 2017;26:227-35.

[13] Simon SL, McWhirter L, Behn CD, Bubar KM, Kaar JL, Pyle L, et al. Morning circadian misalignment is associated with insulin resistance in girls with obesity and polycystic ovarian syndrome. J Clin Endocrinol Metab. 2019;104:3525-34.

[14] Crowley SJ, Suh C, Molina TA, Fogg LF, Sharkey KM, Carskadon MA. Estimating the dim light melatonin onset of adolescents within a 6-h sampling window: the impact of sampling rate and threshold method. Sleep Med. 2016;20:59-66.

[15] Van Reen E, Sharkey KM, Roane BM, Barker D, Seifer R, Raffray T, et al. Sex of college students moderates associations among bedtime, time in bed, and circadian phase angle. J Biol Rhythms. 2013;28:425-31.

[16] McHill AW, Sano A, Hilditch CJ, Barger LK, Czeisler CA, Picard R, et al. Robust stability of melatonin circadian phase, sleep metrics, and chronotype across months in young adults living in real-world settings. J Pineal Res. 2021;70:e12720.

[17] Martin SK, Eastman CI. Sleep logs of young adults with self-selected sleep times predict the dim light melatonin onset. Chronobiol Int. 2002;19:695-707.

[18] Sunde E, Mrdalj J, Pedersen T, Thun E, Bjorvatn B, Gronli J, et al. Role of nocturnal light intensity on adaptation to three consecutive night shifts: a counterbalanced crossover study. Occup Environ Med. 2020;77:249-55.

[19] Stone JE, McGlashan EM, Quin N, Skinner K, Stephenson JJ, Cain SW, et al. The role of light sensitivity and intrinsic circadian period in predicting individual circadian timing. J Biol Rhythms. 2020;35:628-40.

[20] Chakraborty R, Micic G, Thorley L, Nissen TR, Lovato N, Collins MJ, et al. Myopia, or near-sightedness, is associated with delayed melatonin circadian timing and lower melatonin output in young adult humans. Sleep. 2020;44:zsaa208.

[21] Hasler BP, Bruce S, Scharf D, Ngari W, Clark DB. Circadian misalignment and weekend alcohol use in late adolescent drinkers: preliminary evidence. Chronobiol Int. 2019;36:796-810.

[22] Coleman MY, McGlashan EM, Vidafar P, Phillips AJK, Cain SW. Advanced melatonin onset relative to sleep in women with unmedicated major depressive disorder. Chronobiol Int. 2019;36:1373-83.

[23] Nagashima S, Yamashita M, Tojo C, Kondo M, Morita T, Wakamura T. Can tryptophan supplement intake at breakfast enhance melatonin secretion at night? J Physiol Anthropol. 2017;36:20.

[24] Griefahn B. The validity of the temporal parameters of the daily rhythm of melatonin levels as an indicator of morningness. Chronobiol Int. 2002;19:561-77.

[25] Moderie C, Van der Maren S, Paquet J, Dumont M. Home versus laboratory assessments of melatonin production and melatonin onset in young adults complaining of a delayed sleep schedule. J Sleep Res. 2019;29:e12905.

[26] Ruger M, Gordijn MC, Beersma DG, de Vries B, Daan S. Acute and phase-shifting effects of ocular and extraocular light in human circadian physiology. J Biol Rhythms. 2003;18:409-19.

[27] Burgess HJ, Savic N, Sletten T, Roach G, Gilbert SS, Dawson D. The relationship between the dim light melatonin onset and sleep on a regular schedule in young healthy adults. Behav Sleep Med. 2003;1:102-14.

[28] Saxvig IW, Wilhelmsen-Langeland A, Pallesen S, Vedaa O, Nordhus IH, Sørensen E, et al. Objective measures of sleep and dim light melatonin onset in adolescents and young adults with delayed sleep phase disorder compared to healthy controls. J Sleep Res. 2013;22:365-72.

[29] Hisler GC, Rothenberger SD, Clark DB, Hasler BP. Is there a 24-hour rhythm in alcohol craving and does it vary by sleep/circadian timing? Chronobiol Int. 2021;38:109-21.

[30] Sunde E, Pedersen T, Mrdalj J, Thun E, Grønli J, Harris A, et al. Alerting and circadian effects of short-wavelength vs. Long-wavelength narrow-bandwidth light during a simulated night shift. Clocks Sleep. 2020;2:502-22.

[31] Titone MK, McArthur BA, Ng TH, Burke TA, McLaughlin LE, MacMullen LE, et al. Sex and race influence objective and self-report sleep and circadian measures in emerging adults independently of risk for bipolar spectrum disorder. Sci Rep. 2020;10:13731.

[32] Kozaki T, Toda N, Noguchi H, Yasukouchi A. Effects of different light intensities in the morning on dim light melatonin onset. J Physiol Anthropol. 2011;30:97-102.

[33] Flanagan SC, Cobice D, Richardson P, Sittlington JJ, Saunders KJ. Elevated melatonin levels found in young myopic adults are not attributable to a shift in circadian phase. Invest Ophthalmol Vis Sci. 2020;61:45-.

[34] Watson LA, Phillips AJK, Hosken IT, McGlashan EM, Anderson C, Lack LC, et al. Increased sensitivity of the circadian system to light in delayed sleep-wake phase disorder. J Physiol. 2018;596:6249-61.

[35] Van der Maren S, Moderie C, Duclos C, Paquet J, Daneault V, Dumont M. Daily profiles of light exposure and evening use of light-emitting devices in young adults complaining of a delayed sleep schedule. J Biol Rhythms. 2018;33:192-202.

[36] Yasuo S, Iwamoto A, Lee S-i, Ochiai S, Hitachi R, Shibata S, et al. L-serine enhances light-induced circadian phase resetting in mice and humans. J Nutr. 2017;147:2347-55.

[37] Roach GD, Burgess H, Lamond N, Dorrian J, Holmes A. A week of simulated night work delays salivary melatonin onset. J Hum Ergol. 2001;30:255-60.

[38] Fukushige H, Fukuda Y, Tanaka M, Inami K, Wada K, Tsumura Y, et al. Effects of tryptophan-rich breakfast and light exposure during the daytime on melatonin secretion at night. J Physiol Anthropol. 2014;33:33.

[39] Shekleton JA, Rajaratnam SM, Gooley JJ, Van Reen E, Czeisler CA, Lockley SW. Improved neurobehavioral performance during the wake maintenance zone. J Clin Sleep Med. 2013;9:353-62.

[40] Roemer HC, Griefahn B, Kuenemund C, Blaszkewicz M, Gerngroβ H. The reliability of melatonin synthesis as an indicator of the individual circadian phase position. Mil Med. 2003;168:674-8.

[41] Reiter AM, Sargent C, Roach GD. Finding DLMO: estimating dim light melatonin onset from sleep markers derived from questionnaires, diaries and actigraphy. Chronobiol Int. 2020;37:1412-24.

[42] Woelders T, Beersma DGM, Gordijn MCM, Hut RA, Wams EJ. Daily light exposure patterns reveal phase and period of the human circadian clock. J Biol Rhythms. 2017;32:274-86.

[43] Mongrain V, Lavoie S, Selmaoui B, Paquet J, Dumont M. Phase relationships between sleep-wake cycle and underlying circadian rhythms in Morningness-Eveningness. J Biol Rhythms. 2004;19:248-57.

[44] Giménez MC, Beersma DGM, Bollen P, van der Linden ML, Gordijn MCM. Effects of a chronic reduction of short-wavelength light input on melatonin and sleep patterns in humans: Evidence for adaptation. Chronobiol Int. 2014;31:690-7.

[45] Wams EJ, Woelders T, Marring I, van Rosmalen L, Beersma DGM, Gordijn MCM, et al. Linking light exposure and subsequent sleep: a field polysomnography study in humans. Sleep. 2017;40:zsx165.

[46] Gabel V, Maire M, Reichert CF, Chellappa SL, Schmidt C, Hommes V, et al. Effects of artificial dawn and morning blue light on daytime cognitive performance, well-being, cortisol and melatonin levels. Chronobiol Int. 2013;30:988-97.

[47] Sunde E, Pedersen T, Mrdalj J, Thun E, Grønli J, Harris A, et al. Blue-enriched white light improves performance but not subjective alertness and circadian adaptation during three consecutive simulated night shifts. Front Psychol. 2020;11:2172.

[48] Burgess HJ, Eastman CI. The dim light melatonin onset following fixed and free sleep schedules. J Sleep Res. 2005;14:229-37.

[49] Coles ME, Schubert J, Stewart E, Sharkey KM, Deak M. Sleep duration and timing in obsessive-compulsive disorder (OCD): evidence for circadian phase delay. Sleep Med. 2020;72:111-7.

[50] Pullman RE, Roepke SE, Duffy JF. Laboratory validation of an in-home method for assessing circadian phase using dim light melatonin onset (DLMO). Sleep Med. 2012;13:703-6.

[51] Voultsios A, Kennaway DJ, Dawson D. Salivary melatonin as a circadian phase marker: Validation and comparison with plasma melatonin. J Biol Rhythms. 1997;12:457-66.

[52] Solheim B, Olsen A, Kallestad H, Langsrud K, Bjorvatn B, Gradisar M, et al. Cognitive performance in DSWPD patients upon awakening from habitual sleep compared with forced conventional sleep. J Sleep Res. 2019;28:e12730.

[53] Micic G, Lovato N, Ferguson SA, Burgess HJ, Lack L. Circadian tau differences and rhythm associations in delayed sleep-wake phase disorder and sighted non-24-hour sleep-wake rhythm disorder. Sleep. 2020;44:zsaa132.

[54] Wyatt JK, Stepanski EJ, Kirkby J. Circadian phase in delayed sleep phase syndrome: predictors and temporal stability across multiple assessments. Sleep. 2006;29:1075-80.

[55] Thomas JM, Kern PA, Bush HM, McQuerry KJ, Black WS, Clasey JL, et al. Circadian rhythm phase shifts caused by timed exercise vary with chronotype. JCI Insight. 2020;5:e134270.

[56] Domagalik A, Oginska H, Beldzik E, Fafrowicz M, Pokrywka M, Chaniecki P, et al. Long-term reduction of short-wavelength light affects sustained attention and visuospatial working memory with no evidence for a change in circadian rhythmicity. Front Neurosci. 2020;14.

[57] Gabel V, Reichert CF, Maire M, Schmidt C, Schlangen LJM, Kolodyazhniy V, et al. Differential impact in young and older individuals of blue-enriched white light on circadian physiology and alertness during sustained wakefulness. Sci Rep. 2017;7.

[58] McMahon WR, Ftouni S, Phillips AJK, Beatty C, Lockley SW, Rajaratnam SMW, et al. The impact of structured sleep schedules prior to an in-laboratory study: Individual differences in sleep and circadian timing. PLoS One. 2020;15:e0236566.

[59] Burgess HJ, Revell VL, Molina TA, Eastman CI. Human phase response curves to three days of daily melatonin: 0.5 mg versus 3.0 mg. J Clin Endocrinol Metab. 2010;95:3325-31.

[60] Zeeuw Jd, Wisniewski S, Papakonstantinou A, Bes F, Wahnschaffe A, Zaleska M, et al. The alerting effect of the wake maintenance zone during 40 hours of sleep deprivation. Sci Rep. 2018;8:11012.

[61] Suárez A, Núñez F, Rodriguez-Fernandez M. Circadian phase prediction from non-intrusive and ambulatory physiological data. IEEE J Biomed Health Inform. 2021;25:1561-71.

[62] Eastman CI, Martin SK, Hebert M. Failure of extraocular light to facilitate circadian rhythm reentrainment in humans. Chronobiol Int. 2000;17:807-26.

[63] Baron KG, Reid KJ, Kim T, Van Horn L, Attarian H, Wolfe L, et al. Circadian timing and alignment in healthy adults: associations with BMI, body fat, caloric intake and physical activity. Int J Obesity. 2017;41:203-9.

[64] Eastman CI, Molina TA, Dziepak ME, Smith MR. Blacks (African Americans) have shorter free-running circadian periods than whites (Caucasian Americans). Chronobiol Int. 2012;29:1072-7.

[65] Olbrich D, Dittmar M. Older poor-sleeping women display a smaller evening increase in melatonin secretion and lower values of melatonin and core body temperature than good sleepers. Chronobiol Int. 2011;28:681-9.

[66] Weibel J, Lin YS, Landolt HP, Garbazza C, Kolodyazhniy V, Kistler J, et al. Caffeine-dependent changes of sleep-wake regulation: Evidence for adaptation after repeated intake. Prog Neuropsychopharmacol Biol Psychiatry. 2019;99:109851.

[67] Burgess HJ, Fogg LF. Individual differences in the amount and timing of salivary melatonin secretion. PLoS One. 2008;3:e3055.

[68] Revell VL, Kim H, Tseng CY, Crowley SJ, Eastman CI. Circadian phase determined from melatonin profiles is reproducible after 1 wk in subjects who sleep later on weekends. J Pineal Res. 2005;39:195-200.

[69] Park HR, Choi SJ, Jo H, Cho JW, Joo EY. Effects of evening exposure to light from organic light-emitting diodes on melatonin and sleep. J Clin Neurol. 2020;16:401-7.

[70] Wittenbrink N, Ananthasubramaniam B, Munch M, Koller B, Maier B, Weschke C, et al. High-accuracy determination of internal circadian time from a single blood sample. J Clin Invest. 2018;128:3826-39.

[71] Bonmati-Carrion MA, Middleton B, Revell V, Skene DJ, Rol MA, Madrid JA. Circadian phase assessment by ambulatory monitoring in humans: correlation with dim light melatonin onset. Chronobiol Int. 2014;31:37-51.

[72] Krauchi K, Cajochen C, Mori D, Graw P, Wirz Justice A. Early evening melatonin and S-20098 advance circadian phase and nocturnal regulation of core body temperature. Am J Physiol Regul Integr Comp Physiol. 1997;272:R1178-88.

[73] Revell VL, Burgess HJ, Gazda CJ, Smith MR, Fogg LF, Eastman CI. Advancing human circadian rhythms with afternoon melatonin and morning intermittent bright light. J Clin Endocrinol Metab. 2006;91:54-9.

[74] Jo H, Park HR, Choi SJ, Lee SY, Kim SJ, Joo EY. Effects of organic light-emitting diodes on circadian rhythm and sleep. Psychiatry Investig. 2021;18:471-7.

[75] Wright HR, Lack LC, Partridge KJ. Light emitting diodes can be used to phase delay the melatonin rhythm. J Pineal Res. 2001;31:350-5.

[76] Rahman SA, Kayumov L, Tchmoutina EA, Shapiro CM. Clinical efficacy of dim light melatonin onset testing in diagnosing delayed sleep phase syndrome. Sleep Med. 2009;10:549-55.

[77] Rynders CA, Morton SJ, Bessesen DH, Wright Jr. KP, Broussard JL. Circadian rhythm of substrate oxidation and hormonal regulators of energy balance. Obesity. 2020;28:S104-S13.

[78] Goel N. An arousing, musically enhanced bird song stimulus mediates circadian rhythm phase advances in dim light. Am J Physiol Regul Integr Comp Physiol. 2006;291:R822-R7.

[79] van Andel E, Bijlenga D, Vogel SWN, Beekman ATF, Kooij JJS. Effects of chronotherapy on circadian rhythm and ADHD symptoms in adults with attention-deficit/hyperactivity disorder and delayed sleep phase syndrome: a randomized clinical trial. Chronobiol Int. 2020;38:260-9.

[80] Zerbini G, Winnebeck EC, Merrow M. Weekly, seasonal, and chronotype-dependent variation of dim-light melatonin onset. J Pineal Res. 2022;70:e12723.

[81] Van Veen MM, Kooij JJS, Boonstra AM, Gordijn MCM, Van Someren EJW. Delayed circadian rhythm in adults with attention-deficit/hyperactivity disorder and chronic sleep-onset insomnia. Biol Psychiatry. 2010;67:1091-6.

[82] Kawinska A, Dumont M, Selmaoui B, Paquet J, Carrier J. Are modifications of melatonin circadian rhythm in the middle years of life related to habitual patterns of light exposure? J Biol Rhythms. 2005;20:451-60.

[83] Ricketts EJ, Burgess HJ, Montalbano GE, Coles ME, McGuire JF, Thamrin H, et al. Morning light therapy in adults with Tourette's disorder. J Neurol. 2021;269:399-410.

[84] Giménez MC, Hessels M, van de Werken M, de Vries B, Beersma DGM, Gordijn MCM. Effects of artificial dawn on subjective ratings of sleep inertia and dim light melatonin onset. Chronobiol Int. 2010;27:1219-41.

[85] Yoshimura M, Kitamura S, Eto N, Hida A, Katsunuma R, Ayabe N, et al. Relationship between indoor daytime light exposure and circadian phase response under laboratory free-living conditions. Biol Rhythm Res. 2020:1-21.

[86] Baker EK, Richdale AL, Hazi A, Prendergast LA. Assessing the Dim Light Melatonin Onset in adults with autism spectrum disorder and no comorbid intellectual disability. J Autism Dev Disord. 2017;47:2120-37.

[87] Ong JC, Taylor HL, Park M, Burgess HJ, Fox RS, Snyder S, et al. Can circadian dysregulation exacerbate migraines? Headache. 2018;58:1040-51.

[88] Kantermann T, Eastman CI. Circadian phase, circadian period and chronotype are reproducible over months. Chronobiol Int. 2017;35:280-8.

[89] Lovato N, Lack L. Circadian phase delay using the newly developed re-timer portable light device. Sleep Biol Rhythms. 2016;14:157-64.

[90] Wehrens SM, Hampton SM, Skene DJ. Heart rate variability and endothelial function after sleep deprivation and recovery sleep among male shift and non-shift workers. Scand J Work Environ Health. 2012;38:171-81.

[91] Wilson J, IV, Reid KJ, Braun RI, Abbott SM, Zee PC. Habitual light exposure relative to circadian timing in delayed sleep-wake phase disorder. Sleep. 2018;41:zsy166.

[92] Flynn-Evans EE, Shekleton JA, Miller B, Epstein LJ, Kirsch D, Brogna LA, et al. Circadian phase and phase angle disorders in primary insomnia. Sleep. 2017;40:zsx163.

[93] Conroy DA, Hairston IS, Arnedt JT, Hoffmann RF, Armitage R, Brower KJ. Dim light melatonin onset in alcohol-dependent men and women compared with healthy controls. Chronobiol Int. 2012;29:35-42.

[94] Grima NA, Ponsford JL, St. Hilaire MA, Mansfield D, Rajaratnam SM. Circadian melatonin rhythm following traumatic brain injury. Neurorehabil Neural Repair. 2016;30:972-7.

[95] Paul MA, Miller JC, Love RJ, Lieberman H, Blazeski S, Arendt J. Timing light treatment for eastward and westward travel preparation. Chronobiol Int. 2009;26:867-90.

[96] Ruiz FS, Beijamini F, Beale AD, Gonçalves BdSB, Vartanian D, Taporoski TP, et al. Early chronotype with advanced activity rhythms and dim light melatonin onset in a rural population. J Pineal Res. 2020;69:e12675.

[97] Ferguson SA, Kennaway DJ, Baker A, Lamond N, Dawson D. Sleep and circadian rhythms in mining operators: Limited evidence of adaptation to night shifts. Appl Ergon. 2012;43:695-701.

[98] Komarzynski S, Bolborea M, Huang Q, Finkenstadt B, Levi F. Predictability of individual circadian phase during daily routine for medical applications of circadian clocks. JCI Insight. 2019;4:e130423.

[99] Manni R, De Icco R, Cremascoli R, Ferrera G, Furia F, Zambrelli E, et al. Circadian phase typing in idiopathic generalized epilepsy: Dim light melatonin onset and patterns of melatonin secretion—Semicurve findings in adult patients. Epilepsy Behav. 2016;61:132-7.

[100] Paul MA, Gray GW, Lieberman HR, Love RJ, Miller JC, Trouborst M, et al. Phase advance with separate and combined melatonin and light treatment. Psychopharmacology. 2011;214:515-23.

[101] Steele DL, Rajaratnam SM, Redman JR, Ponsford JL. The effect of traumatic brain injury on the timing of sleep. Chronobiol Int. 2005;22:89-105.

[102] Gordijn MC, Beersma DG, Korte HJ, Van-den HRH. Effects of light exposure and sleep displacement on dim light melatonin onset. J Sleep Res. 1999;8:163-74.

[103] Paul MA, Miller JC, Gray GW, Love RJ, Lieberman HR, Arendt J. Melatonin treatment for eastward and westward travel preparation. Psychopharmacology. 2010;208:377-86.

[104] Butler MP, Thosar SS, Smales C, DeYoung PN, Wu H, Hussain MV, et al. Effects of obstructive sleep apnea on endogenous circadian rhythms assessed during relaxed wakefulness; an exploratory analysis. Chronobiol Int. 2020;37:856-66.

[105] Fukuda T, Haraguchi A, Takahashi M, Nakaoka T, Fukazawa M, Okubo J, et al. A randomized, double-blind and placebo-controlled crossover trial on the effect of l-ornithine ingestion on the human circadian clock. Chronobiol Int. 2018;35:1445-55.

[106] Valent F, Mariuz M, Liva G, Verri S, Arlandini S, Vivoli R. Salivary melatonin and cortisol and occupational injuries among Italian hospital workers. Neurol Sci. 2016;37:1613-20.

[107] Ritter P, Soltmann B, Sauer C, Yakac A, Boekstaegers L, Reichard M, et al. Supersensitivity of patients with Bipolar I disorder to light-induced phase delay by narrow bandwidth blue light. Biological Psychiatry Global Open Science. 2022;2:28-35.

[108] Riethmeister V, Bültmann U, Gordijn M, Brouwer S, de Boer M. Investigating daily fatigue scores during two-week offshore day shifts. Appl Ergon. 2018;71:87-94.

[109] Walters JF, Hampton SM, Ferns GA, Skene DJ. Effect of menopause on melatonin and alertness rhythms investigated in constant routine conditions. Chronobiol Int. 2005;22:859-72.

[110] Goel N. Late-night presentation of an auditory stimulus phase delays human circadian rhythms. Am J Physiol Regul Integr Comp Physiol. 2005;289:R209-R16.

[111] Burgess HJ, Kikyo F, Valdespino-Hayden Z, Rizvydeen M, Kimura M, Pollack MH, et al. Do the Morningness-Eveningness questionnaire and Munich ChronoType questionnaire change after morning light treatment? Sleep Sci Pract. 2018;2:12.

[112] Papaioannou I, Twigg GL, Kemp M, Roughton M, Hooper J, Morrell MJ, et al. Melatonin concentration as a marker of the circadian phase in patients with obstructive sleep apnoea. Sleep Med. 2012;13:167-71.

[113] Naismith SL, Hickie IB, Terpening Z, Rajaratnam SW, Hodges JR, Bolitho S, et al. Circadian misalignment and sleep disruption in mild cognitive impairment. J Alzheimers Dis. 2014;38:857-66.

[114] Kim SJ, Lim YC, Suh IB, Lee JH. Disrupted sleep maintenance is associated with altered circadian phase and phase angle in community-dwelling adults. Sleep Med. 2020;73:250-6.

[115] Chinoy ED, Harris MP, Kim MJ, Wang W, Duffy JF. Scheduled evening sleep and enhanced lighting improve adaptation to night shift work in older adults. Occup Environ Med. 2016;73:869-76.

[116] Hoyos CM, Gordon C, Terpening Z, Norrie L, Lewis SJG, Hickie IB, et al. Circadian rhythm and sleep alterations in older people with lifetime depression: a case-control study. BMC Psychiatry. 2020;20:192.

[117] Manni R, Cremascoli R, Perretti C, De Icco R, Picascia M, Ghezzi C, et al. Evening melatonin timing secretion in real life conditions in patients with Alzheimer disease of mild to moderate severity. Sleep Med. 2019;63:122-6.

[118] Bolitho SJ, Naismith SL, Rajaratnam SMW, Grunstein RR, Hodges JR, Terpening Z, et al. Disturbances in melatonin secretion and circadian sleep–wake regulation in Parkinson disease. Sleep Med. 2014;15:342-7.

[119] Figueiro MG, Bierman A, Rea MS. A train of blue light pulses delivered through closed eyelids suppresses melatonin and phase shifts the human circadian system. Nat Sci Sleep. 2013;5:133-41.

[120] Gooneratne NS, Metlay JP, Guo W, Pack FM, Kapoor S, Pack AI. The validity and feasibility of saliva melatonin assessment in the elderly. J Pineal Res. 2003;34:88-94.

[121] Shenshen Y, Minshu W, Qing Y, Yang L, Suodi Z, Wei W. The effect of cataract surgery on salivary melatonin and sleep quality in aging people. Chronobiol Int. 2016;33:1064-72.

Supplementary File 2

The papers reporting plasma DMLO used in the current study.

[1] Mishima K, Okawa M, Shimizu T, Hishikawa Y. Diminished melatonin secretion in the elderly caused by insufficient environmental illumination. J Clin Endocrinol Metab. 2001;86:129-34.

[2] Tozawa T, Mishima K, Satoh K, Echizenya M, Shimizu T, Hishikawa Y. Stability of sleep timing against the melatonin secretion rhythm with advancing age: Clinical implications. J Clin Endocrinol Metab. 2003;88:4689-95.

[3] Sletten TL, Revell VL, Middleton B, Lederle KA, Skene DJ. Age-related changes in acute and phase-advancing responses to monochromatic light. J Biol Rhythms. 2009;24:73-84.

[4] Brown EN, Choe Y, Shanahan TL, Czeisler CA. A mathematical model of diurnal variations in human plasma melatonin levels. Am J Physiol. 1997;272:E506-16.

[5] Duffy JF, Zeitzer JM, Rimmer DW, Klerman EB, Dijk DJ, Czeisler CA. Peak of circadian melatonin rhythm occurs later within the sleep of older subjects. Am J Physiol Endocrinol Metab. 2002;282:E297-E303.

[6] Buxton OM, Lee CW, L'Hermite-Baleriaux M, Turek FW, Van Cauter E. Exercise elicits phase shifts and acute alterations of melatonin that vary with circadian phase. Am J Physiol. 2003;284:R714-R24.

[7] Gooley JJ, Chamberlain K, Smith KA, Khalsa SB, Rajaratnam SM, Van Reen E, et al. Exposure to room light before bedtime suppresses melatonin onset and shortens melatonin duration in humans. J Clin Endocrinol Metab. 2011;96:E463-72.

[8] Luboshitzky R, Ophir U, Nave R, Epstein R, Shen-Orr Z, Herer P. The effect of pyridoxine administration on melatonin secretion in normal men. Neuro Endocrinol Lett. 2002;23:213-7.

[9] Voultsios A, Kennaway DJ, Dawson D. Salivary melatonin as a circadian phase marker: Validation and comparison with plasma melatonin. J Biol Rhythms. 1997;12:457-66.

[10] Santhi N, Lazar AS, McCabe PJ, Lo JC, Groeger JA, Dijk DJ. Sex differences in the circadian regulation of sleep and waking cognition in humans. Proc Natl Acad Sci USA. 2016;113:E2730-9.

[11] Fourtillan JB, Brisson AM, Fourtillan M, Ingrand I, Decourt JP, Girault J. Melatonin secretion occurs at a constant rate in both young and older men and women. Am J Physiol Endocrinol Metab. 2001;280:E11-22.

[12] Baehr EK, Eastman CI, Revelle W, Olson SH, Wolfe LF, Zee PC. Circadian phase-shifting effects of nocturnal exercise in older compared with young adults. Am J Physiol Regul Integr Comp Physiol. 2003;284:R1542-50.

[13] Cagnacci A, Soldani R, Yen SS. Hypothermic effect of melatonin and nocturnal core body temperature decline are reduced in aged women. J Appl Physiol. 1995;78:314-7.

[14] Culnan E, Reid KJ, Zee PC, Crowley SJ, Baron KG. Meal timing relative to DLMO: Associations with BMI and body fat. Sleep Health. 2021;7:339-44.

[15] Rodenbeck A, Huether G, Rüther E, Hajak G. Altered circadian melatonin secretion patterns in relation to sleep in patients with chronic sleep-wake rhythm disorders. J Pineal Res. 1998;25:201-10.

[16] Benloucif S, Guico MJ, Reid KJ, Wolfe LF, L'Hermite-Baleriaux M, Zee PC. Stability of melatonin and temperature as circadian phase markers and their relation to sleep times in humans. J Biol Rhythms. 2005;20:178-88.

[17] Benloucif S, Green K, L'Hermite-Balériaux M, Weintraub S, Wolfe LF, Zee PC. Responsiveness of the aging circadian clock to light. Neurobiol Aging. 2006;27:1870-9.

[18] Allen NH, Smythe PJ, Martin N, Plaskett L, Osola K, Childs P, et al. The effect of temazepam on melatonin secretion and light sensitivity. J Psychopharmacol. 1994;8:94-7.

[19] Dahl K, Avery DH, Lewy AJ, Savage MV, Brengelmann GL, Larsen LH, et al. Dim light melatonin onset and circadian temperature during a constant routine in hypersomnic winter depression. Acta Psychiatr Scand. 1993;88:60-6.

[20] Cagnacci A, Soldani R, Yen SS. Contemporaneous melatonin administration modifies the circadian response to nocturnal bright light stimuli. Am J Physiol. 1997;272:R482-6.

[21] Swanson GR, Gorenz A, Shaikh M, Desai V, Kaminsky T, Berg JVD, et al. Night workers with circadian misalignment are susceptible to alcohol-induced intestinal hyperpermeability with social drinking. Am J Physiol Gastrointest Liver Physiol. 2016;311:G192-G201.

[22] Jones CR, Campbell SS, Zone SE, Cooper F, DeSano A, Murphy PJ, et al. Familial advanced sleep-phase syndrome: A short-period circadian rhythm variant in humans. Nat Med. 1999;5:1062-5.

[23] Williams G, Pirmohamed J, Minors D, Waterhouse J, Buchan I, Arendt J, et al. Dissociation of body-temperature and melatonin secretion circadian rhythms in patients with chronic fatigue syndrome. Clin Physiol. 1996;16:327-37.

[24] Terman JS, Terman M, Lo ES, Cooper TB. Circadian time of morning light administration and therapeutic response in winter depression. Arch Gen Psychiatry. 2001;58:69-75.

[25] Nie J, Zhou T, Chen Z, Dang W, Jiao F, Zhan J, et al. Investigation on entraining and enhancing human circadian rhythm in closed environments using daylight-like LED mixed lighting. Sci Total Environ. 2020;732:139334.

[26] Mäntele S, Otway DT, Middleton B, Bretschneider S, Wright J, Robertson MD, et al. Daily rhythms of plasma melatonin, but not plasma leptin or leptin mRNA, vary between lean, obese and Type 2 diabetic men. PLoS One. 2012;7:e37123.

[27] Steindl PE, Finn B, Bendok B, Rothke S, Zee PC, Blei AT. Disruption of the diurnal rhythm of plasma melatonin in cirrhosis. Ann Int Med. 1995;123:274-7.

[28] Meliska CJ, Martinez LF, Lopez AM, Sorenson DL, Nowakowski S, Parry BL. Relationship of morningness-eveningness questionnaire score to melatonin and sleep timing, body mass index and atypical depressive symptoms in peri- and post-menopausal women. Psychiatry Res. 2011;188:88-95.

[29] Montagnese S, Middleton B, Mani AR, Skene DJ, Morgan MY. On the origin and the consequences of circadian abnormalities in patients with cirrhosis. Am J Gastroenterol. 2010;105:1773-81.

[30] Barger LK, Wright KP, Jr., Hughes RJ, Czeisler CA. Daily exercise facilitates phase delays of circadian melatonin rhythm in very dim light. Am J Physiol Regul Integr Comp Physiol. 2004;286:R1077-R84.

[31] Gooneratne NS, Metlay JP, Guo W, Pack FM, Kapoor S, Pack AI. The validity and feasibility of saliva melatonin assessment in the elderly. J Pineal Res. 2003;34:88-94.

Supplementary File 3.

The source and type of data obtained in the data sets made available by researchers or mined from the literature.

Reference	Individual provider of the data and their institution	Source	DLMO	Age	MEQ
[1]	Stephanie J. Crowley, Rush University Medical Center, USA	Data Share	Y	Y	Y
[2-4]	Erlend Sunde, University of Bergen, Norway,	Data Share	Y	Y	Y
[5]	Thomas Kantermann, University of Applied Science, Essen, Germany	Data Share	Y	Y	Y
[6]	J. Matthew Thomas, University of Kentucky, USA	Data Share	Y	Y	Y
[7]	Andrew Reiter, Central Queensland University, Adelaide, Australia	Data Share	Y	Y	Y
[8]	Ranjay Chakraborty, Flinders University, Australia	Data Share	Y	Y	Y
[9]	Mary Carskadon, Brown University, USA	Data Share	Y	Y	Y
[10]	Emily J. Ricketts, University of California Los Angeles, USA	Data Share	Y	Y	Y
[11]	Takafumi Fukuda, Kirin Company, Ltd., Japan;	Data Share	Y	Y	Y
[12]	Christophe Moderie, McGill University, Canada	Data Share	Y	Y	Y
[13]	Sarah C. Flanagan, Ulster University, Northern Ireland	Data Share	Y	Y	Y
[14]	Nicole Lovato, Flinders University Australia	Data Share	Y	Y	Y
[15]	Raffaele Manni, IRCCS Mondino Foundation, Pavia, Italy.	Data Share	Y	Y	Y
[16, 17]	Elise M McGlashan, Monash University, Australia	Data Share	Y	Y	Y
[18]	Madison K Titone, University of California, San Diego. USA	Data Share	Y	Y	Y
[19]	Meredith Coles, Binghamton University, NY, USA	Data Share	Y	Y	Y
[20]	Kathryn Reid, Northwestern University, USA	Data Share	Y	Y	Y
[21]	Shigekazu Higuchi, Kyushu University, Japan	Data Share	Y	Y	Y
[22]	Gorica Micic, Flinders University, Australia	Data Share	Y	Y	Y
[23, 24]	Tracey Sletten, Monash University, Australia	Data Share	Y	Y	Y

[25]	Camilla Hoyos, University of Sydney, Australia	Data Share	Y	Y	Y
[26]	Kelly Baron, University of Utah, USA	Data Share	Y	Y	Y
[27]	Giulia Zerbini, University of Augsburg, Germany.	Data Share	Y	Y	N
[28]	Eun Yeon Joo, Sungkyunkwan University School of Medicine, Korea	Data Share	Y	Y	N
[29]	Carolin Reichert, University of Basel, Switzerland	Data Share	Y	Y	N
[30]	Beatrix Feigl, Queensland University of Technology, Australia	Data Share	Y	Y	N
[31]	Maria Angeles Rol, University of Murcia, Spain.	Data Share	Y	Y	N
[32]	Stephanie J. Crowley, Rush University Medical Center, USA	Data Share	Y	Y	N
[33]	Monique K. LeBourgeois, University of Colorado Boulder, USA;	Data Share	Y	Y	N
[34]	Aleksandra Domagalik-Pittner, Jagiellonian University, Poland	Data Share	Y	Y	N
[35, 36]	Francis Lévi, Warwick University, United Kingdom	Data Share	Y	Y	N
[37, 38]	Julia Stone, Monash University, Australia	Data Share	Y	Y	N
[39]	Beth Malow, Vanderbilt University School of Medicine, USA	Data Share	Y	Y	N
[40]	Shantha Rajaratnam, Monash University, Australia	Data Share	Y	Y	N
[41]	University of Colorado at Boulder, USA	Paper	Y	Y	N
[42, 43]	Monash University, Australia	Paper	Y	Y	N
[44]	CEMAF, France	Paper	Y	Y	N
[45]	University of South Australia, Australia	Paper	Y	Y	N
[46]	Hôpital du Sacré-Coeur de Montréal, Canada	Paper	Y	Y	N
[47]	Charité Universitätsmedizin Berlin, Germany	Paper	Y	Y	N
[48-50]	Rush University Medical Center, USA	Paper	Y	N	Y
[51]	University of Dortmund, Germany.	Paper	Y	N	Y
[52]	National Institute of Mental Health, Japan,	Paper	Y	N	Y
[53]	University of São Paulo School of Medicine, Brazil	Paper	Y	N	Y

References

[1] Crowley SJ, Van Reen E, LeBourgeois MK, Acebo C, Tarokh L, Seifer R, et al. A longitudinal assessment of sleep timing, circadian phase, and phase angle of entrainment across human adolescence. PLoS One. 2014;9:e112199.

[2] Sunde E, Pedersen T, Mrdalj J, Thun E, Grønli J, Harris A, et al. Alerting and circadian effects of short-wavelength vs. Long-wavelength narrow-bandwidth light during a simulated night shift. Clocks Sleep. 2020;2:502-22.

[3] Sunde E, Pedersen T, Mrdalj J, Thun E, Grønli J, Harris A, et al. Blue-enriched white light improves performance but not subjective alertness and circadian adaptation during three consecutive simulated night shifts. Front Psychol. 2020;11:2172.

[4] Sunde E, Mrdalj J, Pedersen T, Thun E, Bjorvatn B, Gronli J, et al. Role of nocturnal light intensity on adaptation to three consecutive night shifts: a counterbalanced crossover study. Occup Environ Med. 2020;77:249-55.

[5] Kantermann T, Eastman CI. Circadian phase, circadian period and chronotype are reproducible over months. Chronobiol Int. 2017;35:280-8.

[6] Thomas JM, Kern PA, Bush HM, McQuerry KJ, Black WS, Clasey JL, et al. Circadian rhythm phase shifts caused by timed exercise vary with chronotype. JCI Insight. 2020;5:e134270.

[7] Reiter AM, Sargent C, Roach GD. Concordance of chronotype categorisations based on Dim Light Melatonin Onset, the Morningness-Eveningness questionnaire, and the Munich Chronotype Questionnaire. Clocks Sleep. 2021;3.

[8] Chakraborty R, Micic G, Thorley L, Nissen TR, Lovato N, Collins MJ, et al. Myopia, or near-sightedness, is associated with delayed melatonin circadian timing and lower melatonin output in young adult humans. Sleep. 2020;44:zsaa208.

[9] Van Reen E, Sharkey KM, Roane BM, Barker D, Seifer R, Raffray T, et al. Sex of college students moderates associations among bedtime, time in bed, and circadian phase angle. J Biol Rhythms. 2013;28:425-31.

[10] Ricketts EJ, Burgess HJ, Montalbano GE, Coles ME, McGuire JF, Thamrin H, et al. Morning light therapy in adults with Tourette's disorder. J Neurol. 2021;269:399-410.

[11] Fukuda T, Haraguchi A, Takahashi M, Nakaoka T, Fukazawa M, Okubo J, et al. A randomized, double-blind and placebo-controlled crossover trial on the effect of l-ornithine ingestion on the human circadian clock. Chronobiol Int. 2018;35:1445-55.

[12] Moderie C, Van der Maren S, Paquet J, Dumont M. Home versus laboratory assessments of melatonin production and melatonin onset in young adults complaining of a delayed sleep schedule. J Sleep Res. 2019;29:e12905.
[13] Flanagan SC, Cobice D, Richardson P, Sittlington JJ, Saunders KJ. Elevated melatonin levels found in young myopic adults are not attributable to a shift in circadian phase. Invest Ophthalmol Vis Sci. 2020;61:45-.
[14] Lovato N, Lack L. Circadian phase delay using the newly developed re-timer portable light device. Sleep Biol Rhythms. 2016;14:157-64.
[15] Manni R, Cremascoli R, Perretti C, De Icco R, Picascia M, Ghezzi C, et al. Evening melatonin timing secretion in real life conditions in patients with Alzheimer disease of mild to moderate severity. Sleep Med. 2019;63:122-6.
[16] McGlashan EM, Coleman MY, Vidafar P, Phillips AJK, Cain SW. Decreased sensitivity of the circadian system to light in current, but not remitted depression. J Affect Disord. 2019;256:386-92.
[17] Coleman MY, McGlashan EM, Vidafar P, Phillips AJK, Cain SW. Advanced melatonin onset relative to sleep in women with unmedicated major depressive disorder. Chronobiol Int. 2019;36:1373-83.
[18] Titone MK, McArthur BA, Ng TH, Burke TA, McLaughlin LE, MacMullen LE, et al. Sex and race influence objective and self-report sleep and circadian measures in emerging adults independently of risk for bipolar spectrum disorder. Sci Rep. 2020;10:13731.
[19] Coles ME, Schubert J, Stewart E, Sharkey KM, Deak M. Sleep duration and timing in obsessive-compulsive disorder (OCD): evidence for circadian phase delay. Sleep Med. 2020;72:111-7.
[20] Wilson J, IV, Reid KJ, Braun RI, Abbott SM, Zee PC. Habitual light exposure relative to circadian timing in delayed sleep-wake phase disorder. Sleep. 2018;41:zsy166.
[21] Higuchi S, Lee SI, Kozaki T, Harada T, Tanaka I. Late circadian phase in adults and children is correlated with use of high color temperature light at home at night. Chronobiol Int. 2016;33:448-52.
[22] Micic G, Lovato N, Gradisar M, Burgess HJ, Ferguson SA, Kennaway DJ, et al. Nocturnal melatonin profiles in patients with delayed sleep-wake phase disorder and control sleepers. J Biol Rhythms. 2015;30:437-48.
[23] Sletten TL, Segal AY, Flynn-Evans EE, Lockley SW, Rajaratnam SM. Inter-individual differences in neurobehavioural impairment following sleep restriction are associated with circadian rhythm phase. PLoS One. 2015;10:e0128273.

[24] Sletten TL, Vincenzi S, Redman JR, Lockley SW, Rajaratnam SM. Timing of sleep and its relationship with the endogenous melatonin rhythm. Front Neurol. 2010;1:137.
[25] Hoyos CM, Gordon C, Terpening Z, Norrie L, Lewis SJG, Hickie IB, et al. Circadian rhythm and sleep alterations in older people with lifetime depression: a case-control study. BMC Psychiatry. 2020;20:192.
[26] Baron KG, Reid KJ, Kim T, Van Horn L, Attarian H, Wolfe L, et al. Circadian timing and alignment in healthy adults: associations with BMI, body fat, caloric intake and physical activity. Int J Obesity. 2017;41:203-9.
[27] Zerbini G, Winnebeck EC, Merrow M. Weekly, seasonal, and chronotype-dependent variation of dim-light melatonin onset. J Pineal Res. 2022;70:e12723.
[28] Jo H, Park HR, Choi SJ, Lee SY, Kim SJ, Joo EY. Effects of organic light-emitting diodes on circadian rhythm and sleep. Psychiatry Investig. 2021;18:471-7.
[29] Reichert CF, Veitz S, Bühler M, Gruber G, Deuring G, Rehm SS, et al. Wide awake at bedtime? Effects of caffeine on sleep and circadian timing in male adolescents - a randomized crossover trial. Biochem Pharmacol. 2020;191:114283.
[30] Feigl B, Dumpala S, Kerr GK, Zele AJ. Melanopsin cell dysfunction is Involved in sleep disruption in Parkinson's Disease. J Parkinson Dis. 2020;10:1467-76.
[31] Bonmati-Carrion MA, Middleton B, Revell V, Skene DJ, Rol MA, Madrid JA. Circadian phase assessment by ambulatory monitoring in humans: correlation with dim light melatonin onset. Chronobiol Int. 2014;31:37-51.
[32] Misiunaite I, Eastman CI, Crowley SJ. Circadian phase advances in response to weekend morning light in adolescents with short sleep and late bedtimes on school nights. Front Neurosci. 2020;14:99.
[33] LeBourgeois MK, Carskadon MA, Akacem LD, Simpkin CT, Wright KP, Achermann P, et al. Circadian phase and its relationship to nighttime sleep in toddlers. J Biol Rhythms. 2013;28:322-31.
[34] Domagalik A, Oginska H, Beldzik E, Fafrowicz M, Pokrywka M, Chaniecki P, et al. Long-term reduction of short-wavelength light affects sustained attention and visuospatial working memory with no evidence for a change in circadian rhythmicity. Front Neurosci. 2020;14.
[35] Komarzynski S, Bolborea M, Huang Q, Finkenstadt B, Levi F. Predictability of individual circadian phase during daily routine for medical applications of circadian clocks. JCI Insight. 2019;4:e130423.

[36] Lévi F, Komarzynski S, Huang Q, Young T, Ang Y, Fuller C, et al. Tele-monitoring of cancer patients' rhythms during daily life identifies actionable determinants of circadian and sleep disruption. Cancers. 2020;12.
[37] Stone JE, McGlashan EM, Quin N, Skinner K, Stephenson JJ, Cain SW, et al. The role of light sensitivity and intrinsic circadian period in predicting individual circadian timing. J Biol Rhythms. 2020;35:628-40.
[38] Stone JE, Phillips AJK, Chachos E, Hand AJ, Lu S, Carskadon MA, et al. In-person vs. home schooling during the COVID-19 pandemic: Differences in sleep, circadian timing, and mood in early adolescence. J Pineal Res. 2021;71:e12757.
[39] Goldman SE, Alder ML, Burgess HJ, Corbett BA, Hundley R, Wofford D, et al. Characterizing sleep in adolescents and adults with autism spectrum disorders. J Autism Dev Disord. 2017;47:1682-169.
[40] Grima NA, Ponsford JL, St. Hilaire MA, Mansfield D, Rajaratnam SM. Circadian melatonin rhythm following traumatic brain injury. Neurorehabil Neural Repair. 2016;30:972-7.
[41] Akacem LD, Simpkin CT, Carskadon MA, Wright KP, Jr., Jenni OG, Achermann P, et al. The timing of the circadian clock and sleep differ between napping and non-napping toddlers. PLoS One. 2015;10:e0125181.
[42] McGlashan EM, Burns AC, Murray JM, Sletten TL, Magee M, Rajaratnam SMW, et al. The pupillary light reflex distinguishes between circadian and non-circadian delayed sleep phase disorder (DSPD) phenotypes in young adults. PLoS One. 2018;13:e0204621.
[43] McMahon WR, Ftouni S, Phillips AJK, Beatty C, Lockley SW, Rajaratnam SMW, et al. The impact of structured sleep schedules prior to an in-laboratory study: Individual differences in sleep and circadian timing. PLoS One. 2020;15:e0236566.
[44] Fourtillan JB, Brisson AM, Fourtillan M, Ingrand I, Decourt JP, Girault J. Melatonin secretion occurs at a constant rate in both young and older men and women. Am J Physiol Endocrinol Metab. 2001;280:E11-22.
[45] Agostini A, Carskadon MA, Dorrian J, Coussens S, Short MA. An experimental study of adolescent sleep restriction during a simulated school week: changes in phase, sleep staging, performance and sleepiness. J Sleep Res. 2017;26:227-35.
[46] Kawinska A, Dumont M, Selmaoui B, Paquet J, Carrier J. Are modifications of melatonin circadian rhythm in the middle years of life related to habitual patterns of light exposure? J Biol Rhythms. 2005;20:451-60.

[47] Wittenbrink N, Ananthasubramaniam B, Munch M, Koller B, Maier B, Weschke C, et al. High-accuracy determination of internal circadian time from a single blood sample. J Clin Invest. 2018;128:3826-39.
[48] Burgess HJ, Eastman CI. The dim light melatonin onset following fixed and free sleep schedules. J Sleep Res. 2005;14:229-37.
[49] Kantermann T, Sung H, Burgess HJ. Comparing the Morningness-Eveningness Questionnaire and Munich ChronoType Questionnaire to the Dim Light Melatonin Onset. J Biol Rhythms. 2015.
[50] Martin SK, Eastman CI. Sleep logs of young adults with self-selected sleep times predict the dim light melatonin onset. Chronobiol Int. 2002;19:695-707.
[51] Roemer HC, Griefahn B, Kuenemund C, Blaszkewicz M, Gerngroβ H. The reliability of melatonin synthesis as an indicator of the individual circadian phase position. Mil Med. 2003;168:674-8.
[52] Kitamura S, Hida A, Aritake S, Higuchi S, Enomoto M, Kato M, et al. Validity of the Japanese version of the Munich ChronoType Questionnaire. Chronobiol Int. 2014;31:845-50.
[53] Ruiz FS, Beijamini F, Beale AD, Gonçalves BdSB, Vartanian D, Taporoski TP, et al. Early chronotype with advanced activity rhythms and dim light melatonin onset in a rural population. J Pineal Res. 2020;69:e12675.

Supplementary File 4.

The data are the published saliva and plasma DLMO (mean ± SD) from studies which compared younger and older healthy subjects.
Note that in all but 1 study (highlighted) the DLMO of older subjects was earlier than for the younger subjects.

	Saliva/ Plasma	Age	Younger	n	Age	Older	n
[1]	S	26	20.17h	10	69	19.95h	10
[2]	S	30	22.85h ± 1.53	16	50	21.53h ± 1.30	21
[3]	S	25	22.80h ± 1.02	26	65	22.17h ± 0.58	12
[4]	P	27	20.50h ± 0.80	12	70	20.20h ± 0.90	11
[5]	P	23	22.30h ± 1.21	27	69	21.00h ± 1.19	42
[6]	P	23	21.07h ± 0.72	11	66	21.60h ± 1.23	15
[7]	P	21	22.07h ± 1.26	10	71	21.92h ± 0.83	10
[8]	P	23	23.13h ± 1.47	33	68	21.92h ± 1.15	15
[9]	P	27	21.30h ± 1.27	8	58	19.28h ± 1.3	5
[10]	P	29	22.74h ± 1.42	16	67	22.19h ± 1.33	14
[11]	P	27	21.83h ± 1.23	8	64	20.63h ± 0.97	10

References

[1] Olbrich D, Dittmar M. Older poor-sleeping women display a smaller evening increase in melatonin secretion and lower values of melatonin and core body temperature than good sleepers. Chronobiol Int. 2011;28:681-9.

[2] Kawinska A, Dumont M, Selmaoui B, Paquet J, Carrier J. Are modifications of melatonin circadian rhythm in the middle years of life related to habitual patterns of light exposure? J Biol Rhythms. 2005;20:451-60.

[3] Gabel V, Reichert CF, Maire M, Schmidt C, Schlangen LJM, Kolodyazhniy V, et al. Differential impact in young and older individuals of blue-enriched white light on circadian physiology and alertness during sustained wakefulness. Sci Rep. 2017;7.

[4] Fourtillan JB, Brisson AM, Fourtillan M, Ingrand I, Decourt JP, Girault J. Melatonin secretion occurs at a constant rate in both young and older men and women. Am J Physiol Endocrinol Metab. 2001;280:E11-22.

[5] Tozawa T, Mishima K, Satoh K, Echizenya M, Shimizu T, Hishikawa Y. Stability of sleep timing against the melatonin secretion rhythm with advancing age: Clinical implications. J Clin Endocrinol Metab. 2003;88:4689-95.

[6] Sletten TL, Revell VL, Middleton B, Lederle KA, Skene DJ. Age-related changes in acute and phase-advancing responses to monochromatic light. J Biol Rhythms. 2009;24:73-84.

[7] Mishima K, Okawa M, Shimizu T, Hishikawa Y. Diminished melatonin secretion in the elderly caused by insufficient environmental illumination. J Clin Endocrinol Metab. 2001;86:129-34.
[8] Duffy JF, Zeitzer JM, Rimmer DW, Klerman EB, Dijk DJ, Czeisler CA. Peak of circadian melatonin rhythm occurs later within the sleep of older subjects. Am J Physiol Endocrinol Metab. 2002;282:E297-E303.
[9] Cagnacci A, Soldani R, Yen SS. Hypothermic effect of melatonin and nocturnal core body temperature decline are reduced in aged women. J Appl Physiol. 1995;78:314-7.
[10] Benloucif S, Green K, L'Hermite-Balériaux M, Weintraub S, Wolfe LF, Zee PC. Responsiveness of the aging circadian clock to light. Neurobiol Aging. 2006;27:1870-9.
[11] Baehr EK, Eastman CI, Revelle W, Olson SH, Wolfe LF, Zee PC. Circadian phase-shifting effects of nocturnal exercise in older compared with young adults. Am J Physiol Regul Integr Comp Physiol. 2003;284:R1542-50.

Supplementary File 5

The saliva DLMO (mean ± SD) for subjects clinically diagnosed with DSWPD or similar and their mean ages. Also plotted are the n-weighted saliva DLMO for healthy subjects (□). The reference range for healthy individuals is shown as the shaded area.

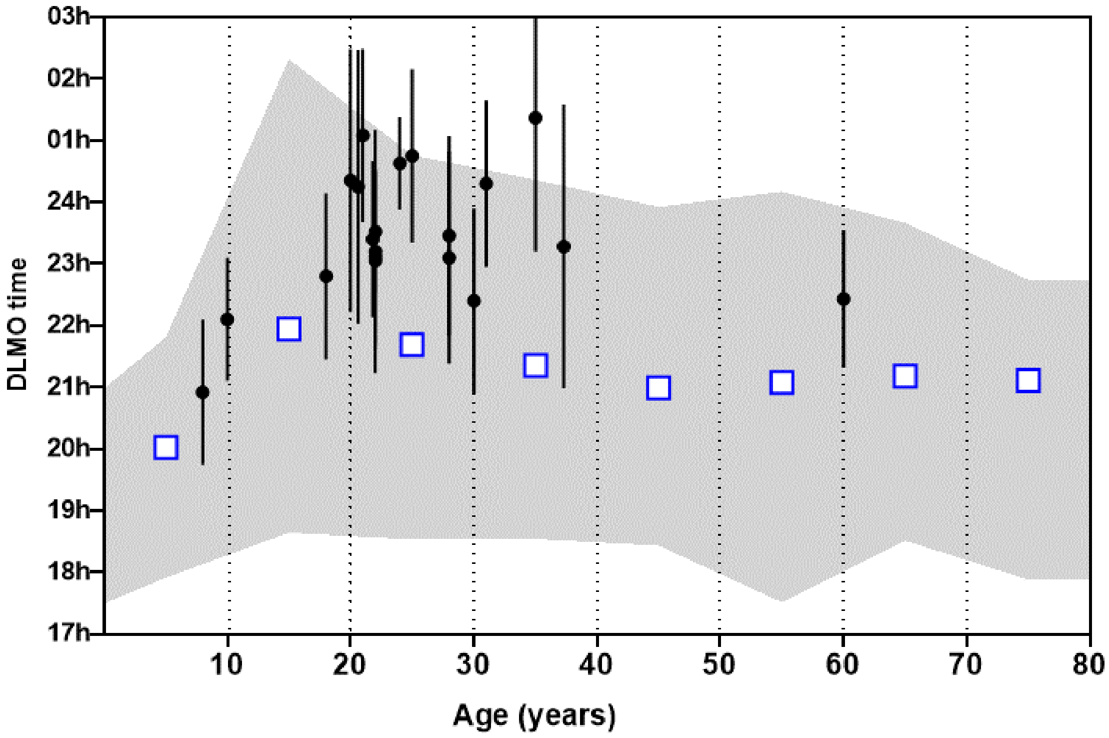

The papers used to create the figure above and a brief description of the criteria used for the diagnosis of DSWPD.

Reference	Criteria for diagnosis of DSPS
[1]	Patients met the ICSD-3 criteria A–E for DSWPD.
[2]	Participants reported at least a 2-hour difference between desired and habitual bedtime, subjective sleep latency at the desired bedtime that suggested sleep onset did not occur until the habitual bedtime.
[3]	Subjects had a habitual bedtime later than midnight with a complaint of a difficulty adapting their sleep timing to standard work/school schedules.
[4]	Habitual Bedtime varied from 00:30 to 04:00 hours (01:36 ± 1:14 hours).
[5]	Diagnostic criteria of the ICSD ; (1) problems falling asleep in the evening, (2) falling asleep after 2 am at least three days a week, (3) ability to sleep until early afternoon, (4) problems waking up in time for school/ studies, (5) early wake-up times associated with extreme daytime sleepiness.
[6]	Had evening-type MEQ, a minimum of 2-h discrepancy between their preferred and current sleep pattern, sleep onset later than 1:00 am but sound sleep quality

[7]	DSWPD patients were eligible if their DLMO time was later than 23.00 h and classified as having 'circadian DSWPD' with DLMO within 30 min before desired bedtime or after desired bedtime.
[8]	Met the ICSD diagnostic criteria for DSPS including complaints of sleep onset insomnia and phase delay of the major sleep episode using two-week sleep diaries. All had subjective complaints of an inability to initiate sleep at a desired clock time which was earlier than their markedly delayed habitual sleep times.
[9]	Met the ICSD criteria for DSWPD as determined by a sleep physician. Controls had a sleep onset no later than 12:30 am, an intermediate MEQ score (42–58).
[10]	Participants met diagnostic criteria for DSPD based on clinical interview by a sleep physician. Participants were classified into phenotype groups based on the relationship between DLMO and desired bedtime (DBT). A circadian DSPD phenotype was defined as having a DLMO time at or after DBT.
[11]	Idiopathic chronic SOI, defined as a sleep onset later than 20:30 hours for children at age 6 years, and for older children 15 min later per year.
[12]	Subjects met ICSD criteria for DSPS. Reported a delay in sleep and wake times relative to the demands of society and an inability to fall asleep at conventional or desired times, as well as difficulty awakening in the morning.
[13]	DSPD subjects had (a) sleep onset later than 01:00 a.m. (b) late sleep offset and great difficulties rising in the morning; (c) if allowed to choose their own sleep-wake rhythm, they exhibited improved sleep quality and duration for age.
[14]	Presence of a misalignment between the participant's usual sleep–wake times and routine schedule, prospectively defined as an obligatory morning commitment requiring waking 1–2.5 h earlier than average wake time at least 1 day per week.
[15]	Diagnostic criteria of the ICSD ; (1) problems falling asleep in the evening, (2) falling asleep after 2 am at least three days a week, (3) ability to sleep until early afternoon, (4) problems waking up in time for school/ studies, (5) early wake-up times associated with extreme daytime sleepiness.
[16]	Idiopathic chronic SOI, defined as a sleep onset later than 20:30 hours for children at age 6 years, and for older children 15 min later per year.
[17]	Diagnosis of DSPD according to the standard criteria of the International Classification of Sleep Disorders, second edition (ICSD-2 and a clinical interview conducted by a psychiatrist-sleep specialist.
[18]	Diagnosed with DSWPD according to the ICSD criteria
[19]	Patients met ICSD criteria
[20]	Patients completed an online questionnaire assessing the time of falling asleep, trouble waking up in the morning, sleep maintenance problems, restless legs, snoring, apnoea and daytime sleepiness. Patients falling asleep late or having trouble waking up at a conventional time in the morning were studied.

References

[1] Solheim B, Olsen A, Kallestad H, Langsrud K, Bjorvatn B, Gradisar M, et al. Cognitive performance in DSWPD patients upon awakening from habitual sleep compared with forced conventional sleep. J Sleep Res. 2019;28:e12730.
[2] Wyatt JK, Stepanski EJ, Kirkby J. Circadian phase in delayed sleep phase syndrome: predictors and temporal stability across multiple assessments. Sleep. 2006;29:1075-80.
[3] Van der Maren S, Moderie C, Duclos C, Paquet J, Daneault V, Dumont M. Daily profiles of light exposure and evening use of light-emitting devices in young adults complaining of a delayed sleep schedule. J Biol Rhythms. 2018;33:192-202.
[4] Moderie C, Van der Maren S, Paquet J, Dumont M. Home versus laboratory assessments of melatonin production and melatonin onset in young adults complaining of a delayed sleep schedule. J Sleep Res. 2019;29:e12905.
[5] Saxvig IW, Wilhelmsen-Langeland A, Pallesen S, Vedaa O, Nordhus IH, Sørensen E, et al. Objective measures of sleep and dim light melatonin onset in adolescents and young adults with delayed sleep phase disorder compared to healthy controls. J Sleep Res. 2013;22:365-72.
[6] Micic G, Lovato N, Ferguson SA, Burgess HJ, Lack L. Circadian tau differences and rhythm associations in delayed sleep-wake phase disorder and sighted non-24-hour sleep-wake rhythm disorder. Sleep. 2020;44:zsaa132.
[7] Watson LA, Phillips AJK, Hosken IT, McGlashan EM, Anderson C, Lack LC, et al. Increased sensitivity of the circadian system to light in delayed sleep-wake phase disorder. J Physiol. 2018;596:6249-61.
[8] Rahman SA, Kayumov L, Tchmoutina EA, Shapiro CM. Clinical efficacy of dim light melatonin onset testing in diagnosing delayed sleep phase syndrome. Sleep Med. 2009;10:549-55.
[9] Wilson J, IV, Reid KJ, Braun RI, Abbott SM, Zee PC. Habitual light exposure relative to circadian timing in delayed sleep-wake phase disorder. Sleep. 2018;41:zsy166.
[10] Murray JM, Sletten TL, Magee M, Gordon C, Lovato N, Bartlett DJ, et al. Prevalence of circadian misalignment and its association with depressive symptoms in delayed sleep phase disorder. Sleep. 2017;40:zsw002-zsw.
[11] Van Der Heijden KB, Smits MG, Van Someren EJW, Boudewijn Gunning W. Prediction of melatonin efficacy by pretreatment dim light melatonin onset in children with idiopathic chronic sleep onset insomnia. J Sleep Res. 2005;14:187-94.

[12] Mundey K, Benloucif S, Harsanyi K, Dubocovich ML, Zee PC. Phase-dependent treatment of delayed sleep phase syndrome with melatonin. Sleep. 2005;28:1271-8.
[13] Danielsson K, Jansson-Fröjmark M, Broman J-E, Markström A. Light therapy with scheduled rise times in young adults with delayed sleep phase disorder: therapeutic outcomes and possible predictors. Behav Sleep Med. 2018;16:325-36.
[14] Sharkey KM, Carskadon MA, Figueiro MG, Zhu Y, Rea MS. Effects of an advanced sleep schedule and morning short wavelength light exposure on circadian phase in young adults with late sleep schedules. Sleep Med. 2011;12:685-92.
[15] Saxvig IW, Wilhelmsen-Langeland A, Pallesen S, Vedaa Ø, Nordhus IH, Bjorvatn B. A randomized controlled trial with bright light and melatonin for delayed sleep phase disorder: Effects on subjective and objective sleep. Chronobiol Int. 2014;31:72-86.
[16] Smits MG, van Stel HF, van der Heijden K, Meijer AM, Coenen AML, Kerkhof GA. Melatonin improves health status and sleep in children with idiopathic chronic sleep-onset insomnia: A randomized placebo-controlled trial. J Am Acad Child Adolesc Psychiatry. 2003;42:1286-93.
[17] Esaki Y, Kitajima T, Ito Y, Koike S, Nakao Y, Tsuchiya A, et al. Wearing blue light-blocking glasses in the evening advances circadian rhythms in the patients with delayed sleep phase disorder: An open-label trial. Chronobiol Int. 2016;33:1037-44.
[18] Reis C, Paiva T. Delayed sleep-wake phase disorder in a clinical population: gender and sub-population diferences. Sleep Science. 2019;12:203-13.
[19] Nagtegaal JE, Kerkhof GA, Smits MG, Swart AC, van-der Meer YG. Delayed sleep phase syndrome: A placebo-controlled cross-over study on the effects of melatonin administered five hours before the individual dim light melatonin onset. J Sleep Res. 1998;7:135-43.
[20] Keijzer H, Smits MG, Peeters T, Looman CWN, Endenburg SC, Gunnewiek JMTK. Evaluation of salivary melatonin measurements for Dim Light Melatonin Onset calculations in patients with possible sleep–wake rhythm disorders. Clin Chim Acta. 2011;412:1616-20.

Supplementary File 6.

The peak plasma melatonin levels in healthy subjects at different ages from 18 papers, where both young and older subjects were studied simultaneously. The median rate of change in melatonin levels was determined and studies divided into those changing at rates above (red lines) and below the median (black lines). Note that the weighted mean levels of the "young" fast declining group was higher than the slowly declining group, but means were similar in the older groups.

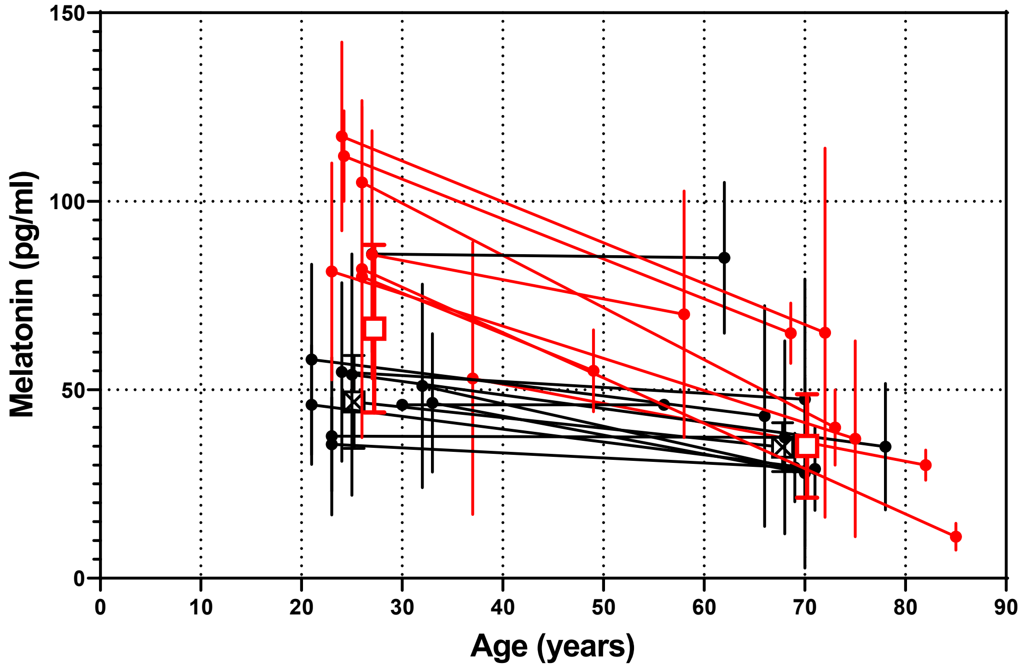

The following table shows the calculated rate of change in melatonin concentration across the ages. The median was 0.51 pg/ml/yr.

Ref	Change (pg/ml/y)	Ref	Change (pg/ml/y)
[1]	0.00	[2]	0.51
[3]	0.01	[4]	0.51
[5]	0.03	[6]	0.59
[7]	0.13	[8]	0.85
[9]	0.16	[10]	1.06
[11]	0.33	[12]	1.09
[13]	0.34	[14]	1.09
[15]	0.36	[16]	1.20
[17]	0.50	[18]	1.38

References for peak melatonin levels with age

[1] Cowen PJ, Bevan JS, Gosden B, Elliott SA. Treatment with beta-adrenoceptor blockers reduces plasma melatonin concentration. Br J Clin Pharmacol. 1985;19:258-60.

[2] Cagnacci A, Soldani R, Yen SS. Hypothermic effect of melatonin and nocturnal core body temperature decline are reduced in aged women. J Appl Physiol. 1995;78:314-7.

[3] Zeitzer JM, Daniels JE, Duffy JF, Klerman EB, Shanahan TL, Dijk DJ, et al. Do plasma melatonin concentrations decline with age? Am J Med. 1999;107:432-6.

[4] Magri F, Locatelli M, Balza G, Molla G, Cuzzoni G, Fioravanti M, et al. Changes in endocrine circadian rhythms as markers of physiological and pathological brain aging. Chronobiol Int. 1997;14:385-96.

[5] Claustrat B, Chazot G, Brun J, Jordan D, Sassolas G. A chronobiological study of melatonin and cortisol secretion in depressed subjects: plasma melatonin, a biochemical marker in major depression. Biol Psychiatry. 1984;19:1215-28.

[6] Sugaya K, Nishijima S, Oda M, Owan T, Miyazato M, Ogawa Y. Biochemical and body composition analysis of nocturia in the elderly. Neurourol Urodyn. 2008;27:205-11.

[7] Tozawa T, Mishima K, Satoh K, Echizenya M, Shimizu T, Hishikawa Y. Stability of sleep timing against the melatonin secretion rhythm with advancing age: Clinical implications. J Clin Endocrinol Metab. 2003;88:4689-95.

[8] Coevorden Av, Mockel J, Laurent E, Kerkhofs M, L'Hermite-Baleriaux M, Decoster C, et al. Neuroendocrine rhythms and sleep in aging men. Am J Physiol Endocrinol Metab. 1991;260:E651-E61.

[9] Fourtillan JB, Brisson AM, Fourtillan M, Ingrand I, Decourt JP, Girault J. Melatonin secretion occurs at a constant rate in both young and older men and women. Am J Physiol Endocrinol Metab. 2001;280:E11-22.

[10] Nair NP, Hariharasubramanian N, Pilapil C, Isaac I, Thavundayil JX. Plasma melatonin--an index of brain aging in humans? Biol Psychiatry. 1986;21:141-50.

[11] Uchida K, Okamoto N, Ohara K, Morita Y. Daily rhythm of serum melatonin in patients with dementia of the degenerate type. Brain Res. 1996;717:154-9.

[12] Touitou Y, Fevre M, Lagoguey M, Carayon A, Bogdan A, Reinberg A, et al. Age- and mental health-related circadian rhythms of plasma levels of melatonin, prolactin, luteinizing hormone and follicle- stimulating hormone in man. J Endocrinol. 1981;91:467-75.

[13] Mishima K, Okawa M, Shimizu T, Hishikawa Y. Diminished melatonin secretion in the elderly caused by insufficient environmental illumination. J Clin Endocrinol Metab. 2001;86:129-34.

[14] Hajak G, Rodenbeck A, Bandelow B, Friedrichs S, Heuther G, Ruther E. Nocturnal plasma melatonin levels after flunitrazepam administration in healthy subjects. Eur Neuropsychopharmacol. 1996;6:149-53.

[15] Ferrari E, Magri F, Dori D, Migliorati G, Nescis T, Molla G, et al. Neuroendocrine correlates of the aging brain in humans. Neuroendocrinology. 1995;61:464-70.

[16] Iguchi H, Kato KI, Ibayashi H. Age-dependent reduction in serum melatonin concentrations in healthy human subjects. J Clin Endocrinol Metab. 1982;55:27-9.

[17] Bartsch C, Bartsch H, Flüchter S-H, Attanasio A, Gupta D. Evidence for modulation of melatonin secretion in men with benign and malignant tumors of the prostate: relationship with the pituitary hormones. J Pineal Res. 1985;2:121-32.

[18] Thomas DR, Miles A. Melatonin secretion and age. Biol Psychiatry. 1989;25:365-7.

Supplementary File 7.

Correlation of MEQ versus DLMO from individual data sets or mined from the literature.

Ref	Slope	R squared	P value	Deviation from zero?	n	Mean age (y)
[1]	-3.755	0.333	<0.0001	Significant	52	24
[2]	-4.365	0.481	<0.0001	Significant	59	30.5
[3]	-4.097	0.318	<0.0001	Significant	80	22
[4]	-4.525	0.325	<0.0001	Significant	98	26.8
[5]	-3.516	0.507	0.001	Significant	19	31.7
[6, 7]	-3.010	0.343	0.001	Significant	31	22.5
[8]	-2.803	0.232	<0.001	Significant	397	18.8
[9]	-2.103	0.232	0.006	Significant	31	22
[10]	-3.617	0.232	0.013	Significant	26	20
[11]	-1.475	0.075	0.025	Significant	67	21.9
[12]	-2.901	0.376	0.026	Significant	13	22.1
[13]	-4.162	0.199	0.026	Significant	25	21.4
[14]	-1.576	0.086	0.026	Significant	58	27.9
[15]	-3.036	0.329	0.032	Significant	14	23.9
[16]	-1.900	0.063	0.035	Significant	71	23.1
[17, 18]	-3.200	0.258	0.053	Not Significant	15	21.2
[19]	-1.682	0.058	0.080	Not Significant	54	36.4
[20]	2.688	0.258	0.092	Not Significant	12	33.6
[21]	-3.211	0.134	0.103	Not Significant	21	39.9
[22]	-1.998	0.098	0.112	Not Significant	27	23.3
[23]	8.508	0.056	0.112	Not Significant	46	21.4
[24]	-0.933	0.061	0.167	Not Significant	33	32.4
[25]	-3.762	0.089	0.322	Not Significant	13	67.5
[26]	1.270	0.031	0.354	Not Significant	30	21.6
[27]	-0.326	0.005	0.735	Not Significant	27	65.7
[28]	-0.311	0.005	0.772	Not Significant	18	23.8
[29]	0.536	0.007	0.803	Not Significant	12	32.3
[30]	-0.119	0.000	0.871	Not Significant	93	13.4
[31]	-0.145	0.000	0.944	Not Significant	15	9.7
Combined	-2.163	0.147	<0.0001	Significant	1457	

n = the number of data points in the individual studies

References

[1] Thomas JM, Kern PA, Bush HM, McQuerry KJ, Black WS, Clasey JL, et al. Circadian rhythm phase shifts caused by timed exercise vary with chronotype. JCI Insight. 2020;5:e134270.

[2] Eastman CI, Tomaka VA, Crowley SJ. Circadian rhythms of European and African-Americans after a large delay of sleep as in jet lag and night work. Sci Rep. 2016;6:36716.

[3] Roemer HC, Griefahn B, Kuenemund C, Blaszkewicz M, Gerngroβ H. The reliability of melatonin synthesis as an indicator of the individual circadian phase position. Mil Med. 2003;168:674-8.

[4] Baron KG, Reid KJ, Kim T, Van Horn L, Attarian H, Wolfe L, et al. Circadian timing and alignment in healthy adults: associations with BMI, body fat, caloric intake and physical activity. Int J Obesity. 2017;41:203-9.

[5] Ricketts EJ, Burgess HJ, Montalbano GE, Coles ME, McGuire JF, Thamrin H, et al. Morning light therapy in adults with Tourette's disorder. J Neurol. 2021;269:399-410.

[6] Sletten TL, Segal AY, Flynn-Evans EE, Lockley SW, Rajaratnam SM. Inter-individual differences in neurobehavioural impairment following sleep restriction are associated with circadian rhythm phase. PLoS One. 2015;10:e0128273.

[7] Sletten TL, Vincenzi S, Redman JR, Lockley SW, Rajaratnam SM. Timing of sleep and its relationship with the endogenous melatonin rhythm. Front Neurol. 2010;1:137.

[8] Van Reen E, Sharkey KM, Roane BM, Barker D, Seifer R, Raffray T, et al. Sex of college students moderates associations among bedtime, time in bed, and circadian phase angle. J Biol Rhythms. 2013;28:425-31.

[9] Chakraborty R, Micic G, Thorley L, Nissen TR, Lovato N, Collins MJ, et al. Myopia, or near-sightedness, is associated with delayed melatonin circadian timing and lower melatonin output in young adult humans. Sleep. 2020;44:zsaa208.

[10] Martin SK, Eastman CI. Sleep logs of young adults with self-selected sleep times predict the dim light melatonin onset. Chronobiol Int. 2002;19:695-707.

[11] Titone MK, McArthur BA, Ng TH, Burke TA, McLaughlin LE, MacMullen LE, et al. Sex and race influence objective and self-report sleep and circadian measures in emerging adults independently of risk for bipolar spectrum disorder. Sci Rep. 2020;10:13731.

[12] Moderie C, Van der Maren S, Paquet J, Dumont M. Home versus laboratory assessments of melatonin production and melatonin onset in young adults complaining of a delayed sleep schedule. J Sleep Res. 2019;29:e12905.

[13] Sunde E, Mrdalj J, Pedersen T, Thun E, Bjorvatn B, Gronli J, et al. Role of nocturnal light intensity on adaptation to three consecutive night shifts: a counterbalanced crossover study. Occup Environ Med. 2020;77:249-55.
[14] Burgess HJ, Eastman CI. The dim light melatonin onset following fixed and free sleep schedules. J Sleep Res. 2005;14:229-37.
[15] Coles ME, Schubert J, Stewart E, Sharkey KM, Deak M. Sleep duration and timing in obsessive-compulsive disorder (OCD): evidence for circadian phase delay. Sleep Med. 2020;72:111-7.
[16] Reiter AM, Sargent C, Roach GD. Concordance of chronotype categorisations based on Dim Light Melatonin Onset, the Morningness-Eveningness questionnaire, and the Munich Chronotype Questionnaire. Clocks Sleep. 2021;3.
[17] McGlashan EM, Coleman MY, Vidafar P, Phillips AJK, Cain SW. Decreased sensitivity of the circadian system to light in current, but not remitted depression. J Affect Disord. 2019;256:386-92.
[18] Coleman MY, McGlashan EM, Vidafar P, Phillips AJK, Cain SW. Advanced melatonin onset relative to sleep in women with unmedicated major depressive disorder. Chronobiol Int. 2019;36:1373-83.
[19] Ruiz FS, Beijamini F, Beale AD, Gonçalves BdSB, Vartanian D, Taporoski TP, et al. Early chronotype with advanced activity rhythms and dim light melatonin onset in a rural population. J Pineal Res. 2020;69:e12675.
[20] Wilson J, IV, Reid KJ, Braun RI, Abbott SM, Zee PC. Habitual light exposure relative to circadian timing in delayed sleep-wake phase disorder. Sleep. 2018;41:zsy166.
[21] Fukuda T, Haraguchi A, Takahashi M, Nakaoka T, Fukazawa M, Okubo J, et al. A randomized, double-blind and placebo-controlled crossover trial on the effect of l-ornithine ingestion on the human circadian clock. Chronobiol Int. 2018;35:1445-55.
[22] Sunde E, Pedersen T, Mrdalj J, Thun E, Grønli J, Harris A, et al. Blue-enriched white light improves performance but not subjective alertness and circadian adaptation during three consecutive simulated night shifts. Front Psychol. 2020;11:2172.
[23] Flanagan SC, Cobice D, Richardson P, Sittlington JJ, Saunders KJ. Elevated melatonin levels found in young myopic adults are not attributable to a shift in circadian phase. Invest Ophthalmol Vis Sci. 2020;61:45-.
[24] Kantermann T, Eastman CI. Circadian phase, circadian period and chronotype are reproducible over months. Chronobiol Int. 2017;35:280-8.

[25] Manni R, Cremascoli R, Perretti C, De Icco R, Picascia M, Ghezzi C, et al. Evening melatonin timing secretion in real life conditions in patients with Alzheimer disease of mild to moderate severity. Sleep Med. 2019;63:122-6.
[26] Sunde E, Pedersen T, Mrdalj J, Thun E, Grønli J, Harris A, et al. Alerting and circadian effects of short-wavelength vs. Long-wavelength narrow-bandwidth light during a simulated night shift. Clocks Sleep. 2020;2:502-22.
[27] Hoyos CM, Gordon C, Terpening Z, Norrie L, Lewis SJG, Hickie IB, et al. Circadian rhythm and sleep alterations in older people with lifetime depression: a case-control study. BMC Psychiatry. 2020;20:192.
[28] Micic G, Lovato N, Gradisar M, Burgess HJ, Ferguson SA, Kennaway DJ, et al. Nocturnal melatonin profiles in patients with delayed sleep-wake phase disorder and control sleepers. J Biol Rhythms. 2015;30:437-48.
[29] Lovato N, Lack L. Circadian phase delay using the newly developed re-timer portable light device. Sleep Biol Rhythms. 2016;14:157-64.
[30] Crowley SJ, Van Reen E, LeBourgeois MK, Acebo C, Tarokh L, Seifer R, et al. A longitudinal assessment of sleep timing, circadian phase, and phase angle of entrainment across human adolescence. PLoS One. 2014;9:e112199.
[31] Higuchi S, Lee SI, Kozaki T, Harada T, Tanaka I. Late circadian phase in adults and children is correlated with use of high color temperature light at home at night. Chronobiol Int. 2016;33:448-52.

Supplementary File 8

Papers reporting melatonin levels in older normal sleepers and those diagnosed with primary insomnia.

References 1-10 report no difference, while references 11-13 conclude that insomniacs produce less melatonin. The numbers of citations were collected from the Web of Science in July 2022

Ref	Institution	Conclusions	Year of publication	Citations	Citations /year
[1]	University of South Australia and University of Adelaide, Australia.	"No significant differences in melatonin excretion were observed between controls and insomniacs."	1998	24	1
[2]	Department of Psychiatry, University of California, San Diego.	"Total daily excretion of 6-SMT was not significantly related to total sleep time, wake-within-sleep or sleep complaints."	1998	37	1.54
[3]	Department of Psychiatry, University of California, San Diego.	"Data indicate that low melatonin production may not be an important factor in insomnia among the elderly."	1998	33	1.38
[4]	University of South Australia and University of Adelaide, Australia.	"Although insomniacs showed a greater amount of wakefulness, less sleep in total, and lower sleep efficiency, no significant group differences were observed in any of the melatonin parameters."	1999	23	1
[5]	Clinics Hospital, Montevideo, Uruguay and University of Buenos Aires, Argentina	"No strict correlation was found between prior 6-sulphatoxymelatonin levels in urine and subsequent sleep improvement after receiving melatonin."	2000	45	1.96
[6]	Sanatorios Los Arroyos e IPAM, Laboratorio de Analisis Bioquımicos e Inmunologicos, Rosario, Argentina; and University of Buenos Aires, Argentina	"The urinary excretion of aMT6s before starting administration of melatonin correlated negatively and significantly with age, but not with the intensity of the sleep disorder or the outcome of treatment,"	2001	53	2.52

[7]	Waitemata Health Ltd, Auckland, New Zealand, University of Auckland, New Zealand and University of Surrey, UK	"No difference in mean aMT6s 24 hour or total night excretory levels, or night/day ratios."	2001	26	1.24
[8]	Waitemata Health Ltd, Auckland, New Zealand, University of Auckland, New Zealand and University of Surrey, UK	"There was a significant difference between the groups in self-reported sleep quality indicators at entry, but no difference in melatonin secretion."	2003	44	2.32
[9]	Department of Psychiatry and Psychotherapy, Charite–Universitatsmedizin, Berlin, Germany	"Data failed to show any age-controlled partial correlation between 6-sulphatoxymelatonin (aMT6s) parameters and PSG parameters in either of the two groups."	2007	9	0.6
[10]	CPS Research, UK., University of Glasgow, UK. Neurim Pharmaceuticals Ltd. Israel, Tel-Aviv, University, Israel.	"A low melatonin excretion level, regardless of age, is not useful in predicting response to Prolonged Release Melatonin in insomnia".	2010	82	6.83
[11]	Technion Israel Institute of Technology, Israel, Neurim Pharmaceuticals Ltd, Israel, Tel Aviv University and Maccabi Pharmaceuticals Ltd, Israel	"Melatonin deficiency seems to be a key variable in the incidence of sleep disorders in elderly people, and melatonin replacement therapy may prove beneficial."	1994	242	8.64
[12]	Wolfson Medical Center, Bat-Yam, Israel, Neurim Pharmaceuticals Ltd, Maccabi Medical Care and Health Fund, and Tel-Aviv University.	"Peak excretion of the main melatonin metabolite 6-sulphatoxymelatonin during the night was lower than normal and/or delayed in comparison with non-insomniac elderly people."	1995	373	13.81

[13]	Centre du Sommeil, Hotel-Dieu de Paris, Paris, France; Neurim Pharmaceuticals Ltd., Israel; and Tel Aviv University, Israel.	Low nocturnal melatonin production is associated with insomnia in patients aged 55 years or older and identifies patients who are somewhat more likely to respond to melatonin replacement. Melatonin replacement therapy may improve sleep quality by treating an underlying deficiency in the endogenous production of this sleep-regulating hormone.	2004	117	6.5

[1] Lushington K, Lack L, Kennaway DJ, Rogers N, van-den HC, Dawson D. 6-Sulfatoxymelatonin excretion and self-reported sleep in good sleeping controls and 55-80-year-old insomniacs. J Sleep Res. 1998;7:75-83.

[2] Kripke DF, Elliot JA, Youngstedt SD, Smith JS. Melatonin: marvel or marker? AnnMed. 1998;30:81-7.

[3] Youngstedt SD, Kripke DF, Elliott JA. Melatonin excretion is not related to sleep in the elderly. J Pineal Res. 1998;24:142-5.

[4] Lushington K, Dawson D, Kennaway DJ, Lack L. The relationship between 6-sulphatoxymelatonin and polysomnographic sleep in good sleeping controls and wake maintenance insomniacs, aged 55-80 years. J Sleep Res. 1999;8:57-64.

[5] Monti JM, Alvarino F, Cardinali D, Savio I, Pintos A. Polysomnographic study of the effect of melatonin on sleep in elderly patients with chronic primary insomnia. Arch Gerontol Geriatr. 1999;28:85-98.

[6] Siegrist C, Benedetti C, Orlando A, Beltran JM, Tuchscherr L, Noseda CM, et al. Lack of changes in serum prolactin, FSH, TSH, and estradiol after melatonin treatment in doses that improve sleep and reduce benzodiazepine consumption in sleep-disturbed, middle-aged, and elderly patients. J Pineal Res. 2001;30:34-42.

[7] Baskett JJ, Wood PC, Broad JB, Duncan JR, English J, Arendt J. Melatonin in older people with age-related sleep maintenance problems: a comparison with age matched normal sleepers. Sleep. 2001;24:418-24.

[8] Baskett JJ, Broad JB, Wood PC, Duncan JR, Pledger MJ, English J, et al. Does melatonin improve sleep in older people? A randomised crossover trial. Age Ageing. 2003;32:164-70.

[9] Mahlberg R, Kunz D. Melatonin excretion levels and polysomnographic sleep parameters in healthy subjects and patients with sleep-related disturbances. Sleep Med. 2007;8:512-6.

[10] Wade AG, Ford I, Crawford G, McConnachie A, Nir T, Laudon M, et al. Nightly treatment of primary insomnia with prolonged release melatonin for 6 months: a randomized placebo controlled trial on age and endogenous melatonin as predictors of efficacy and safety. BMC Med. 2010;8:18.

[11] Haimov I, Laudon M, Zisapel N, Souroujon M, Nof D, Shlitner A, et al. Sleep disorders and melatonin rhythms in elderly people. Br Med J. 1994;309:167X.

[12] Garfinkel D, Laudon M, Nof D, Zisapel N. Improvement of sleep quality in elderly people by controlled- release melatonin. Lancet. 1995;346:541-4.

[13] Leger D, Laudon M, Zisapel N. Nocturnal 6-sulfatoxymelatonin excretion in insomnia and its relation to the response to melatonin replacement therapy. Am J Med. 2004;116:91-5.
